# Metabolomic and transcriptomic landscape of the bovine uterine lumen during the second week of the estrous cycle

**DOI:** 10.1186/s40104-026-01466-z

**Published:** 2026-07-09

**Authors:** Abdul Waheed, Cecilia Constantino Rocha, Alexandra Bennett Bastien, Mariangela Bueno Cordeiro Maldonado, Nadia Ashrafi, Romana A. Mimi, Stewart F. Graham, Francisco Peñagaricano, Mario Binelli

**Affiliations:** 1 Department of Animal Sciences, University of Florida, Gainesville, FL USA; 2 Institute of Continuing Education & Extension, Cholistan University of Veterinary and Animal Sciences, Bahawalpur, Punjab Pakistan; 3 Division of Animal Sciences, Southwest Research, Extension, and Education Center, University of Missouri, Mt Vernon, MO USA; 4 Metabolomics Department, Corewell Health Research Institute, Royal Oak, MI USA; 5 Corewell Health East William Beaumont University Hospital, Royal Oak, MI USA; 6 Oakland University-William Beaumont School of Medicine, Rochester, MI USA; 7 Department of Animal and Dairy Sciences, University of Wisconsin-Madison, Madison, WI USA

**Keywords:** Cattle, Metabolome, Transcriptome, Uterine lumen

## Abstract

**Background:**

In cattle, the second week after estrus encompasses critical changes in uterine function. The endometrium will either prepare for the release of luteolytic pulses of prostaglandin-F2 alpha or for the support of an eventual pregnancy. We hypothesized that concentrations of amino acids and lipids in the uterine luminal fluid (ULF), and the gene expression in luminal epithelial cells (LE), change across the second week of the estrous cycle. The objective was to compare amino acid and lipid concentrations in ULF and target gene expression in LE 7 (D7), 10 and 14 d after estrus. The ULF and LE samples were collected from the uterine body of five primiparous, non-lactating, cyclic *Bos indicus*-influenced crossbred cows using a cytological brush on each day after synchronized estrus. Targeted metabolomics of ULF was performed using mass spectrometry, and gene expression in LE was assessed using RNA sequencing. Data were analyzed by uni- and multivariate statistics.

**Results:**

Multivariate analyses separated D7, D10, and D14, with amino acid metabolism and lipid biosynthesis as enriched pathways. Luminal concentrations of amino acids (e.g., arginine, histidine) and lipids (e.g., CE(17:1), PC aa C34:1) increased from D7 to D10, but from D10 to D14 concentrations of amino acids (e.g., glutamine, glutamic acid) decreased while that of lipids (e.g., CE(18:2), SM C16:0) continued to increase. Transcriptomic profiling revealed temporal regulation of 186 amino acid-related and 133 lipid-related genes. Between D7 and D10, there was increased expression of genes for oxidative phosphorylation and extracellular secretion pathways supporting secretory capacity, while decreased expression of genes in arginine-proline metabolism and solute carrier-mediated transport pathways promoted luminal metabolite accumulation. From D10 to D14, there was increased expression of genes in fatty acid elongation and sphingolipid metabolism pathways likely driving lipid synthesis and amino acid catabolism, while decreased expression of genes in protein digestion/absorption and PI3K/AKT signaling pathways reduced nutrient export to the lumen.

**Conclusions:**

ULF composition and LE gene expression changed markedly during the second week of the estrous cycle. It is plausible that the dynamic shifts in amino acid and lipid metabolites in the uterine lumen reflect changing responses to sex steroids. The influence of such changes on embryo development and pregnancy success in cattle warrants investigation.

**Supplementary Information:**

The online version contains supplementary material available at 10.1186/s40104-026-01466-z.

## Background

In cattle, after ovulation, as the corpus luteum develops, plasmatic concentrations of progesterone rise. The binding of progesterone to its receptor (PGR) quickly downregulates endometrial expression of estrogen receptor (ESR1) and oxytocin receptor (OXTR) and 7 to 10 d after ovulation downregulates the expression of its own receptor [1–3]. Downregulation occurs primarily in luminal epithelium, superficial glands, and superficial stroma [4]. The downregulation of ESR1 and subsequently PGR causes the endometrium to shift from a proliferative to a differentiation and secretory phenotype [5, 6]. This shift regulates endometrial gene expression dramatically, as documented earlier [7–10].

**Fig. 1:**
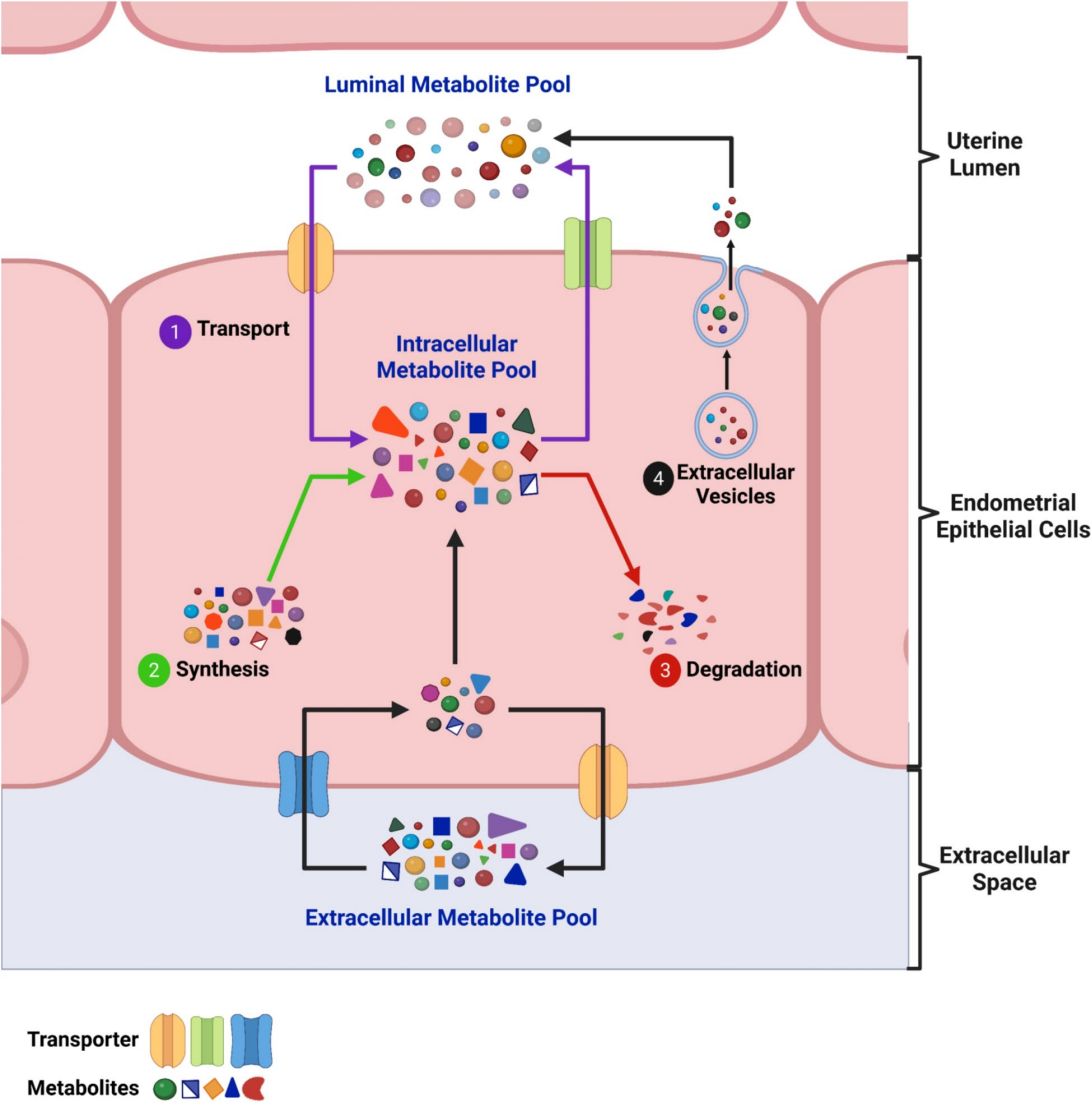
Hypothetical mechanisms regulating metabolite availability in the uterine lumen. The composition of the luminal metabolite pool is determined by the flux of molecules to and from the endometrial epithelial cells via mechanisms such as membrane transporter (1) and extracellular vesicle-mediated transport (4). Metabolites that compose the intracellular metabolite pool present in endometrial epithelial cells originate from multiple cellular processes, such as (1) transport to and from the luminal metabolite pool and to and from the extracellular metabolite pool, via membrane bound specific transporters; (2) synthesis reflects intracellular metabolic activity that generates or interconverts metabolites by intracellular metabolic pathways; (3) degradation represents reduced metabolite availability by degradation into byproducts or utilization within the epithelial cells. Extracellular region has metabolite contribution from the blood as well. Collectively, these proposed processes likely act in concert to regulate metabolic fluxes among the lumen, epithelial cells, and extracellular space, ultimately shaping the uterine luminal environment

The changing endometrial transcriptome is expected to affect the composition of the uterine luminal fluid. Indeed, the luminal environment is unique and different from the peripheral blood. Concentrations of molecules in the uterine luminal fluid are different than those in blood plasma in cows [11, 12], sheep [13], and mares [14]. This is evidence that there is local regulation of molecules in the uterus, likely to support pregnancy. Indeed, we showed that perturbation in the uterine lumen leads to the loss of pregnancy [15]. The uterine luminal fluid contains a blend of molecules such as amino acids, enzymes, carbohydrates, growth factors, hormones, ions, lipids, and proteins [16–18]. Composition of luminal fluid can be modulated by ovarian steroid hormones [17, 19, 20], uterine cellular activity [21], and the stage of the estrous cycle [22]. Molecules detected in the uterine lumen originate from the blood, endometrial glands, luminal epithelium, and stroma [23], and are selectively transported in and out of that compartment [24]. The metabolite concentration in the uterine lumen and endometrial epithelial cells can possibly be regulated by different hypothetical processes like transport, synthesis, and degradation (Fig. 1). It is documented that the temporal changes in the expression of nutrient transporters in the endometrium reflected their concentrations in the uterine lumen [19].

Amino acids [25–27] and lipids [20, 28–30] represent two biochemical classes of metabolites present in the uterine luminal fluid that likely play structural and regulatory roles associated with uterine functions such as preparing for the release of luteolytic pulses of prostaglandin F2-alpha and supporting conceptus growth. Amino acids provide mitogenic signals, direct protein synthesis, act as energy substrates, control the uterine oxidative status, regulate gene expression and modulate the immune system [26, 31]. Lipids, including cholesterol and its esters, contribute to membrane and organelles biogenesis, provide energy support, and act as precursors for bioactive molecules including prostaglandins and steroids that regulate uterine function [29]. The balance and availability of these metabolites are likely central to conceptus elongation, implantation, and maternal recognition of pregnancy in cattle. Indeed, nutrient deficiency in uterine fluid induced mouse embryonic diapause [32].

Previous studies have examined the uterine metabolite concentrations during the preimplantation period [11, 17, 20, 27, 33] by collecting uterine samples from slaughtered or surgically catheterized animals. Here, we expanded the previous knowledge on the temporal dynamics of the composition of the uterine milieu during the second week of the estrous cycle by collecting samples from the uterine lumen of the same animals on each time point with a non-invasive sample collection technique, and by adopting a multi-omics approach including metabolomics and transcriptomics analysis from the same samples. The overarching hypothesis is that endometrial function changes temporally during the second week after estrus to support embryo development. The experimental hypothesis is that concentrations of amino acids and lipids in the uterine luminal fluid, and the gene expression in luminal epithelial cells change across the second week of the estrous cycle. The objective of this study was to characterize the temporal changes in amino acid and lipid concentrations in uterine luminal fluid and the expression of amino acid and lipid-related genes in luminal epithelium among D7, D10, and D14.

## Methods

### Animals

Animal procedures were approved by the Institutional Animal Care and Use Committee (IACUC) of the University of Florida under protocol No. IACUC-202200000055. The research was conducted at the Beef Research Unit (coordinates: 29.743259, −82.264860) of the University of Florida in Gainesville, USA. *Bos indicus* influenced (∼20% of Brahman genetics), primiparous, non-lactating, cyclic, non-pregnant cows (*n* = 5; ~ 2 years old; body weight: 471 ± 72 kg) were enrolled in this study. The cows were maintained under grazing conditions in a 24.3-hectare paddock, primarily featuring Bahiagrass pasture (*Paspalum notatum*), and were supplemented with 20% crude protein range cubes (Wapole Feed, Florida, USA). They also had free access to water and mineral supplements (Furst-McNess, Illinois, USA).

### Estrus detection and criteria for inclusion in the experiment

Estrous cycles were synchronized by the administration of two injections of PGF_2_α (dinoprost tromethamine 25 mg, Lutalyse, Zoetis Animal Health, Florham Park, USA) 14 d apart. After the second injection, animals were fitted with an estrus detection patch (ESTROTECT, Rockway Inc, USA). Estrous detection by ESTROTECT was performed by the visual observation and scoring of ESTROTECT on cows twice a day at 8:00 and 17:00. The visual observation was performed from 24 to 96 h after the administration of the second PGF_2_α injection and lasted at least 30 min. A trained operator observed the mounting behavior and scored each cow according to the proportion of the silver coating removed from the ESTROTECT. The day of estrus was denominated D0 of the estrous cycle.

### Cytobrush collection and sample processing

Uterine luminal sampling was carried out for each cow on D7, D10, and D14 of the estrous cycle, following the procedures described by Cardoso et al. [34] and further detailed by Silva et al. [35]. A total of fifteen samples (*n* = 5 from D7, *n* = 5 from D10, and *n* = 5 from D14) were analyzed for metabolomics. Briefly, caudal epidural anesthesia was administered using 60 mg of 2% Lidocaine (VetOne^®^, Boise, ID, USA) to ensure the cows remained comfortable during the process. The tip of an 8-inch cytobrush (Viamed Ltd., Miami Lakes, FL, USA) was then attached to the end of a standard artificial insemination (AI) gun. This setup was encased in a disposable AI sheath and further protected with a sanitary sheath to maintain sterility. The entire apparatus was carefully inserted through the cervix, with the tip of the AI gun positioned just cranial to the cervical internal os. Once at the uterine body, the cytobrush was carefully exposed and gently rotated five times to collect a sample from the uterine lumen. After collection, the cytobrush was carefully retracted back into the AI sheath to prevent contact with other parts of the reproductive tract, and the whole device was gently removed from the cow. Outside the cow, the cytobrush was detached from the apparatus and immediately placed into a microcentrifuge tube filled with 1 mL of phosphate-buffered saline. The samples were kept on ice and processed within 1 h of collection to minimize molecular degradation. For processing, cells were separated from the brush by vortexing the tube for 1 min. Then, the brush was taken out, and the liquid contents were centrifuged at 400 × *g* for 7 min to separate the cells and any debris. The supernatant, containing the soluble components, was aspirated and placed in a fresh tube, on ice until further processing. The cytobrush was returned to the microcentrifuge tube with the remaining cell pellet, quickly frozen using liquid nitrogen, and stored at −80 °C to prepare for RNA extraction and transcriptomic studies. The supernatant was drawn through a 40-µm pipette tip cell strainer (FLOWMI™ CELL STRAINER, Bel-Art H-B Instrument, Wayne, NJ, USA) to filter out remaining debris. The filtrate was next transferred into a 1 mL syringe (BD, Franklin Lakes, NJ, USA) and slowly passed through a sterile 0.22 µm PVDF syringe filter (Fisherbrand, Dublin, Ireland) to further remove cells or particles. The resulting filtrate, hereafter denominated uterine fluid, was then snap-frozen and kept at −80 °C, ready for subsequent metabolomic analysis.

### RNA extraction

Total RNA extraction of the luminal epithelial cells collected from the cytobrush was performed using the RNeasy Mini Kit (Qiagen #74104, Germantown, MD, USA) following the manufacturer's instructions. Briefly, cellular material in the pellet and adhered to the cytological brush was thawed in ice. The pellet was resuspended in 500 μL of buffer RLT supplemented with 1% of *β*-mercaptoethanol and disrupted by vortexing at maximum speed for 2 min. The brush was discarded, and the lysate was transferred to a QIAshredder spin column (Qiagen #79654) placed in a 2 mL collection tube and centrifuged at 14,000 × *g* for 2 min at room temperature for removal of cellular debris and mucus. The filtrate from the column was mixed with 500 µL of 70% ethanol and the content was loaded into the RNeasy spin column. Samples were subjected to on-column DNase treatment using the RNase-free DNase set (Qiagen #79254). Total RNA was eluted with 30 µL of RNase-free water. Concentration and purity of RNA in extracts were evaluated using spectrophotometry (NanoDrop, Microvolume Spectrophotometer and Fluorometer, Waltham, MA, USA). RNA samples were stored at −80 °C for future library preparation and RNA sequencing.

### Library preparation and RNA sequencing

RNA samples were sequenced by Novogene Corporation. The quality control of the RNA samples was performed before library generation, and RNA Integrity Number (RIN) ranged from 5.7 to 9.3 [see Additional file 2 (Table S1)]. Sequencing libraries were constructed using the RNA-NEBNext Ultra RNA Library Prep Kit for Illumina (New England Biolabs, Ipswich, MA, USA) and the sequencing was performed using NovaSeq 6000 (Illumina, Sacramento, CA, USA). Of 15 total samples, two samples did not pass the quality control, before library preparation due to reduced RIN or reduced RNA concentration and were excluded [see Additional file 2 (Table S1)]. A total of 13 samples (*n* = 4 from D7, *n* = 5 from D10, and *n* = 4 from D14) were successfully extracted, processed and sequenced.

### RNA sequencing: read mapping and counting

Quality of the sequencing reads was assessed using the software FastQC (version 0.11.7, Babraham Bioinformatics, Cambridge, UK). Both read trimming and adapter removal were performed using the software Trim Galore (version 0.4.4, Babraham Bioinformatics) using the following parameters: --paired, --length 50, --clip_R1 15, --clip_R2 15, --three_prime_clip_R1 5, and --three_prime_clip_R2 5. After editing, sequencing reads were mapped to the bovine reference genome ARS-UCD1.2 using the software Hisat2 (v2.1.0) [36]. The number of reads that mapped to each annotated gene in the reference genome ARS-UCD1.2 was estimated using htseq-count (v0.6.1p1) with the option intersection-nonempty [37].

### RNA sequencing: data analysis

Differentially expressed genes between time points were detected using the R package edgeR [38]. This R package uses the trimmed mean of M-values as a normalization method, estimates tagwise negative binomial dispersion parameters using an empirical Bayes procedure, and fits generalized linear models followed by likelihood ratio tests for detecting differentially expressed genes. Here, samples were collected across time from the same groups of cows, so the likelihood ratio test detected genes that were differentially expressed between time points, adjusting simultaneously for differences between cows.* P*-values were adjusted for multiple testing using the FDR procedure. Based on these results, eight different patterns of gene expression (Patterns A to H) were identified. Further four lists of genes were generated: genes that increased expression between D7 and D10 (pattern I, encompassing patterns A, B, and D), decreased expression between D7 and D10 (pattern J, encompassing patterns D, E, and G), increased expression between D10 and D14 (pattern K, encompassing patterns A, C, and H) and, decreased expression between D10 and D14 (pattern L, encompassing patterns E, F, and H). The details of genes in each new pattern (I–L) are given in Additional file 1 (Tables S11–S14).

### Overrepresentation analysis

Genes in patterns I to L were subjected to functional enrichment analysis using EnrichKit [39] which integrates data from five different databases: Gene Ontology (GO), InterPro, Kyoto Encyclopedia of Genes and Genomes (KEGG), Medical Subject Headings (MeSH), and Reactome. False Discovery Rate was controlled at FDR ≤ 0.1. For each of lists I to L, the top 10 enriched terms from each database (50 terms total per list) were selected to evaluate gene expression trends during the second week of the estrous cycle, [see Additional file 1 (Tables S15–S18)].

### Targeted analysis of enriched pathways

Lists of enriched pathways generated from each of the five databases for each of the four lists (I–L) were screened for specific keywords relevant to metabolism and transport of amino acids and lipids. Keywords were: amino acids, alanine, arginine, asparagine, aspartic acid, glutamine, glutamic acid, glycine, serine, tryptophan, valine, lipids, ceramides, cholesterol, sphingolipids, fatty acids, and transporters. Genes associated with the terms containing these keywords and with an unadjusted *P* < 0.1 were selected for inspection of their potential biological relevance and functional contribution to metabolism and transport of amino acids and lipids (Tables 2, 3, 4 and 5). The summary for the steps of data generation from untargeted gene patterns to targeted keywords is provided in Additional file 3 (Fig. S1).

### Targeted analysis of select genes

The summary for the following steps of data generation is provided in Additional file 3 (Fig. S2). Lists were generated separately for amino acid- and lipid-related genes. For amino acids, we initially generated a list of 457 genes potentially involved in transport, catabolism, and synthesis by searching the GO database (accessible at https://www.geneontology.org/) with the following search filters in “Genes and gene product” tab: Organism – *Bos taurus*; Source – UniProtKB; Direct annotation – amino acid, amino acids, alanine, arginine, asparagine, aspartic acid, cysteine, glutamine, glutamic acid, glycine, histidine, isoleucine, lysine, methionine, phenylalanine, proline, serine, threonine, tryptophan, tyrosine, valine, metabolic process, synthesis, biosynthetic process, catabolic process, transport and transporter. This obtained gene list was filtered for duplicates and overlapping genes, which resulted in a GO-derived list of 264 unique genes. Subsequently, we cross-referenced this gene with our RNA-sequencing (RNA-seq) dataset and by removing 35 genes that were not present in the RNA-seq dataset, we maintained 229 genes common to both the GO-derived list and the RNA-seq dataset. Next, we pasted the IDs of these 229 genes on Database for Annotation, Visualization, and Integrated Discovery (DAVID) Functional Annotation Bioinformatics Microarray Analysis (https://davidbioinformatics.nih.gov/), and we selected all genes from 15 pathways with a Benjamini–Hochberg *p*-value 0.008 or smaller related to amino acid transport, catabolism, and synthesis (a total of 660 genes). All the selected genes from the selected pathways were filtered for duplicate and overlapping genes (212 genes removed) and then cross-referenced with the previously generated initial GO-derived list (156 genes removed), resulting in a DAVID-derived list of 292 unique genes. From this list, 13 genes were not present in our RNA-seq dataset, therefore, they were removed. The DAVID-derived list of 279 final genes and the GO-derived list of 229 genes were combined to generate a final compiled list of 508 genes related to amino acid transport, catabolism, and synthesis. From these 508 genes, the expression of 186 genes was significantly affected by time (*P* < 0.05) in our study. Finally, these genes were each classified according to their main function (i.e., transport, catabolism, and synthesis) and direction of change over time (i.e., increased, decreased, or unchanged; Table 6, Additional file 1; Table S19). We adopted the same approach for lipids and generated a final list of 613 genes related to lipid processes, from which the expression of 133 genes changed across time in the current study (Table 7 and Additional file 1; Table S20).

### Metabolomics: data generation

Targeted metabolomics analyses of the uterine fluid were performed using MxP**®** Quant 500 kit (biocrates Life Sciences AG, Innsbruck, Austria). This assay measured the concentration (in micromoles) of 630 metabolites from 26 biochemical classes. LC-MS grade Acetonitrile, Methanol, Isopropyl alcohol, and Formic acid (≥ 99.0% purity) were purchased from Fisher Scientific (Hanover Park, IL, USA), while Ethanol, Pyridine, and Phenylisothiocyanate were sourced from Sigma Aldrich (St. Louis, MO, USA). Milli-Q Water for the aqueous mobile phase was supplied by EMD Millipore (Billerica, MA, USA). Uterine fluid samples were prepped following biocrates Life Sciences (Innsbruck, Austria) guidelines. Samples and calibration standards were thawed on ice, vortexed, and centrifuged as directed. Calibration standards and quality control (QC) standards were mixed in 100 µL H_2_O at 1,200 r/min for 15 min. On a 96-well plate, we pipetted 20 µL uterine fluid, 10 µL each of standard, QCs, and phosphate buffer, then the plate was dried under nitrogen for 30 min. All samples were derivatized with phenylisothiocyanate (PITC) at room temperature for 60 min and dried again for 60 min. Extracts were shaken with 5 mmol/L ammonium acetate in methanol for 30 min, centrifuged at 500 × *g* for 2 min, and diluted 1:1 with H_2_O for the liquid chromatography (LC). For the flow injection analysis (FIA), 20 µL of uterine fluid was mixed with 480 µL of kit solvent, and 10 µL of QC extract with 490 µL of kit solvent were prepared in a separate plate. Both plates were sealed, mixed at 600 r/min for 10 min, and loaded into a thermostatted autosampler. Sample extracts were analyzed on a Waters I-class UPLC coupled with a Waters Xevo-TQ-S (Milford, MA, USA) using an MxP Quant 500 C18 column with guard and precolumn mixer. The mobile phase (A: H_2_O + 0.2% formic acid; B: MeCN + 0.2% formic acid) ran at 0.8 mL/min, gradient 0–100% B over 4.50 min, up to 1.00 mL/min at 100% B for 30 s, then back to initial conditions for 70 s. Gradients were 5.80 min, with negative mode differing in %B from 2.00–4.50 min; injections were 5 µL (positive) and 15 µL (negative), with wash solvent as H₂O:MeOH:MeCN:IPA. FIA-MS/MS used the Q500 kit’s isocratic method (290 mL MeOH + FIA additives) at 0.03 mL/min, 20 µL injections for both modes, and data were extracted using biocrates’ WebIDQsoftware. Three QC samples (low, mid, high) were provided by biocrates.

### Metabolomics: data curation

Prior to analyses, data were log-transformed to achieve normal distribution. Metabolites presenting more than 50% of values below the limit of detection were excluded. For the remaining metabolites, values below the limit of detection were substituted by one-fifth of the lowest concentration detected for that metabolite. A series of created variables, such as pre-defined sums and ratios of specific metabolites, was also generated. The formulas used to calculate created variables are provided in Additional file 1 (Table S2). For ratios, formulas were adjusted by excluding metabolites with missing values from the numerator, whereas variables were not calculated if metabolites were missing in the denominator.

### Metabolomics: multivariate analysis

**Fig. 2:**
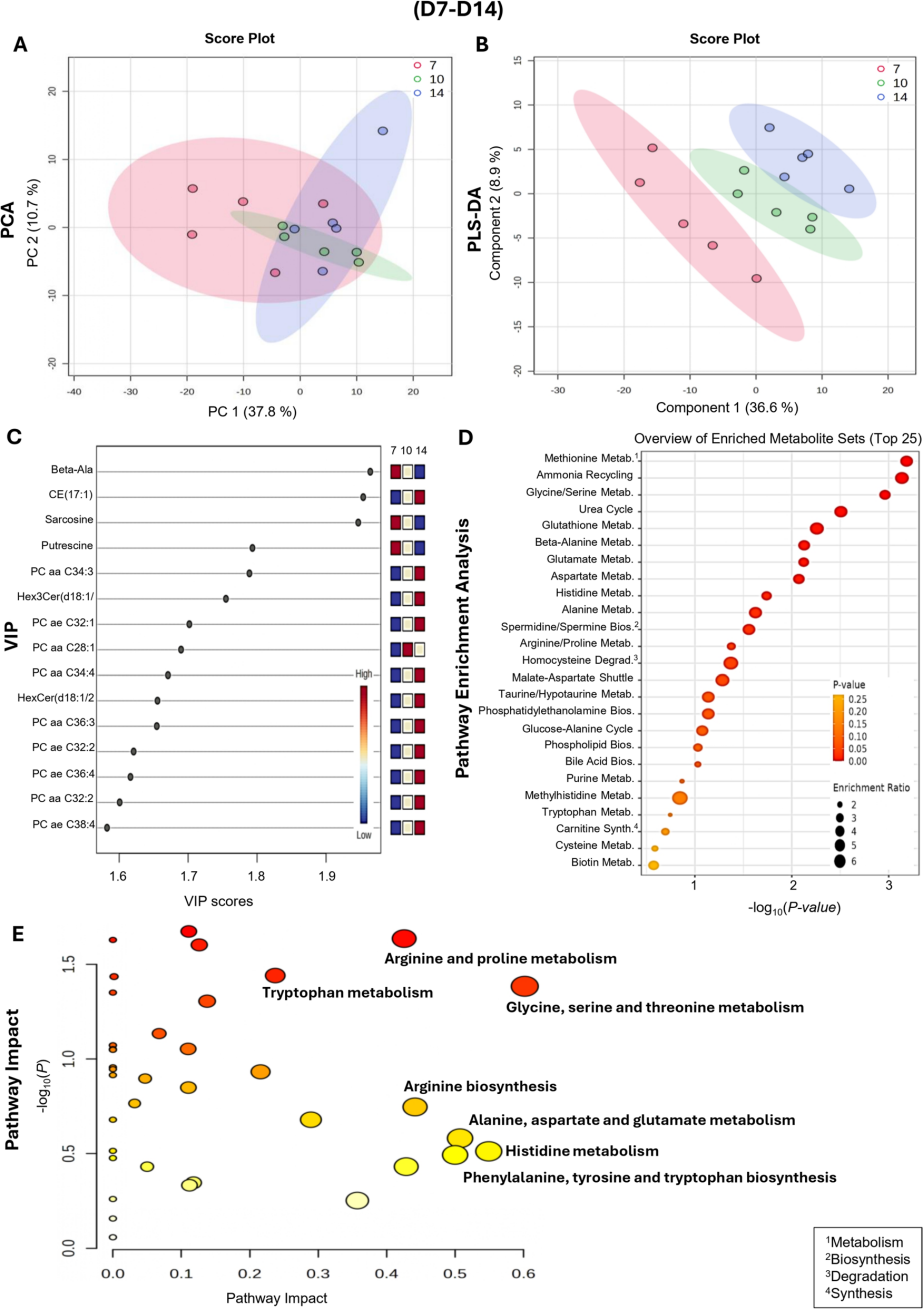
Multivariate and functional enrichment analyses of the uterine luminal metabolome on D7, D10, and D14. **A** Principal component analysis (PCA) score plot of all metabolites for D7–D14. **B** Partial least squares-discriminant analysis (PLS-DA) for D7–D14. **C** Top 15 variable importance in projection (VIP) plot for D7–D14. **D** Quantitative Enrichment Analysis highlighting the metabolic pathways that were enriched in D7–D14. The dots are colored based on the pathway *P*-value and relative size is based on the enrichment ratio. **E** Pathway impact analysis of differentially abundant pathways for D7–D14. Each dot represents an enriched metabolic pathway; dots are colored based on their *P*-value and relative size is based on the pathway impact value

**Fig. 3:**
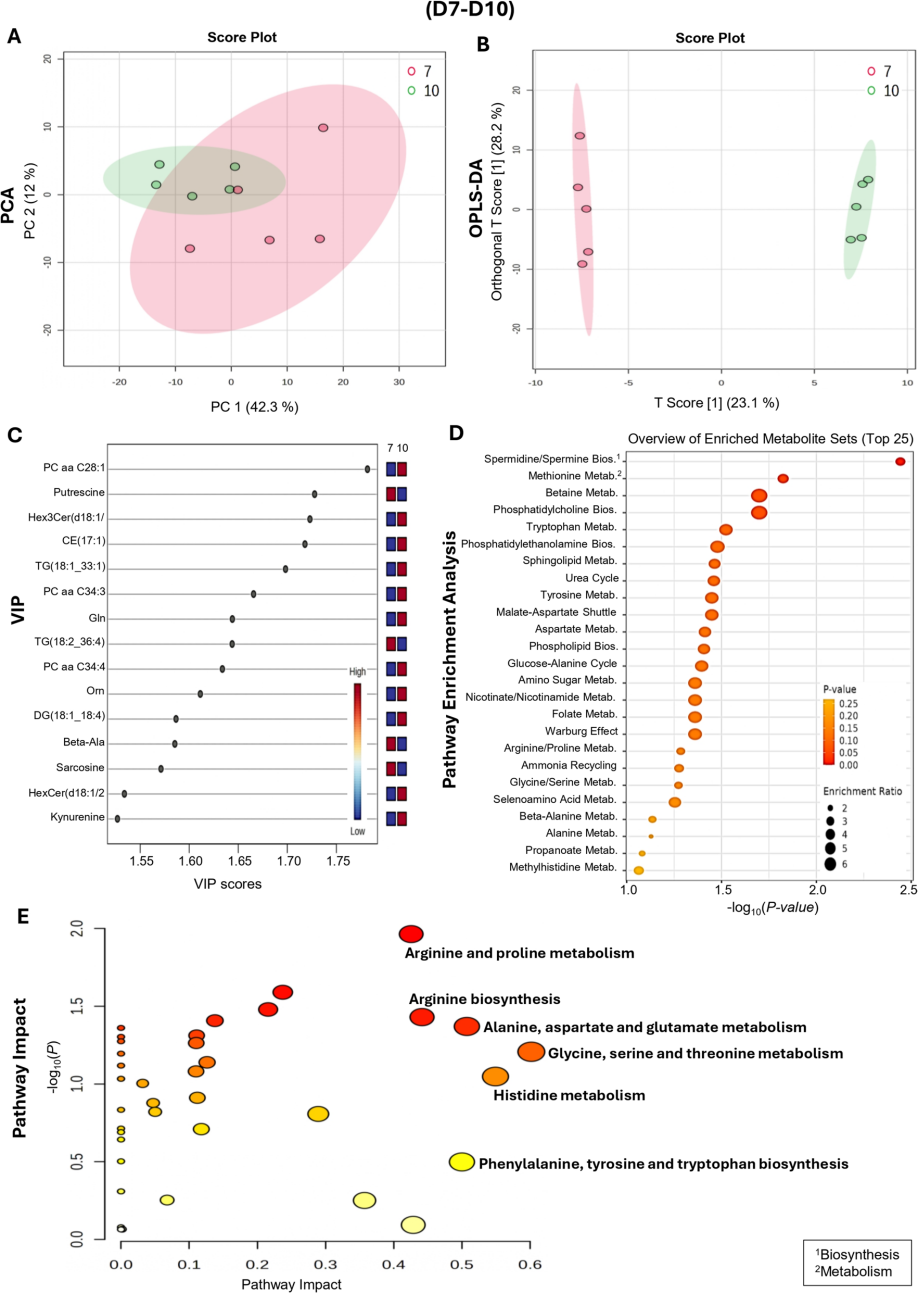
Multivariate and functional enrichment analyses of the uterine luminal metabolome on D7 and D10. **A** Principal component analysis (PCA) score plot of all metabolites for D7–D10. **B** Orthogonal partial least-squares discriminant analysis (OPLS-DA) for D7–D10. **C** Top 15 variable importance in projection (VIP) plot for D7–D10. **D** Quantitative Enrichment Analysis highlighting the metabolic pathways that were enriched in D7–D10. The dots are colored based on the pathway *P*-value and relative size is based on the enrichment ratio. **E** Pathway impact analysis of differentially abundant pathways for D7–D10. Each dot represents an enriched metabolic pathway; dots are colored based on their *P*-value and relative size is based on the pathway impact value

**Fig. 4:**
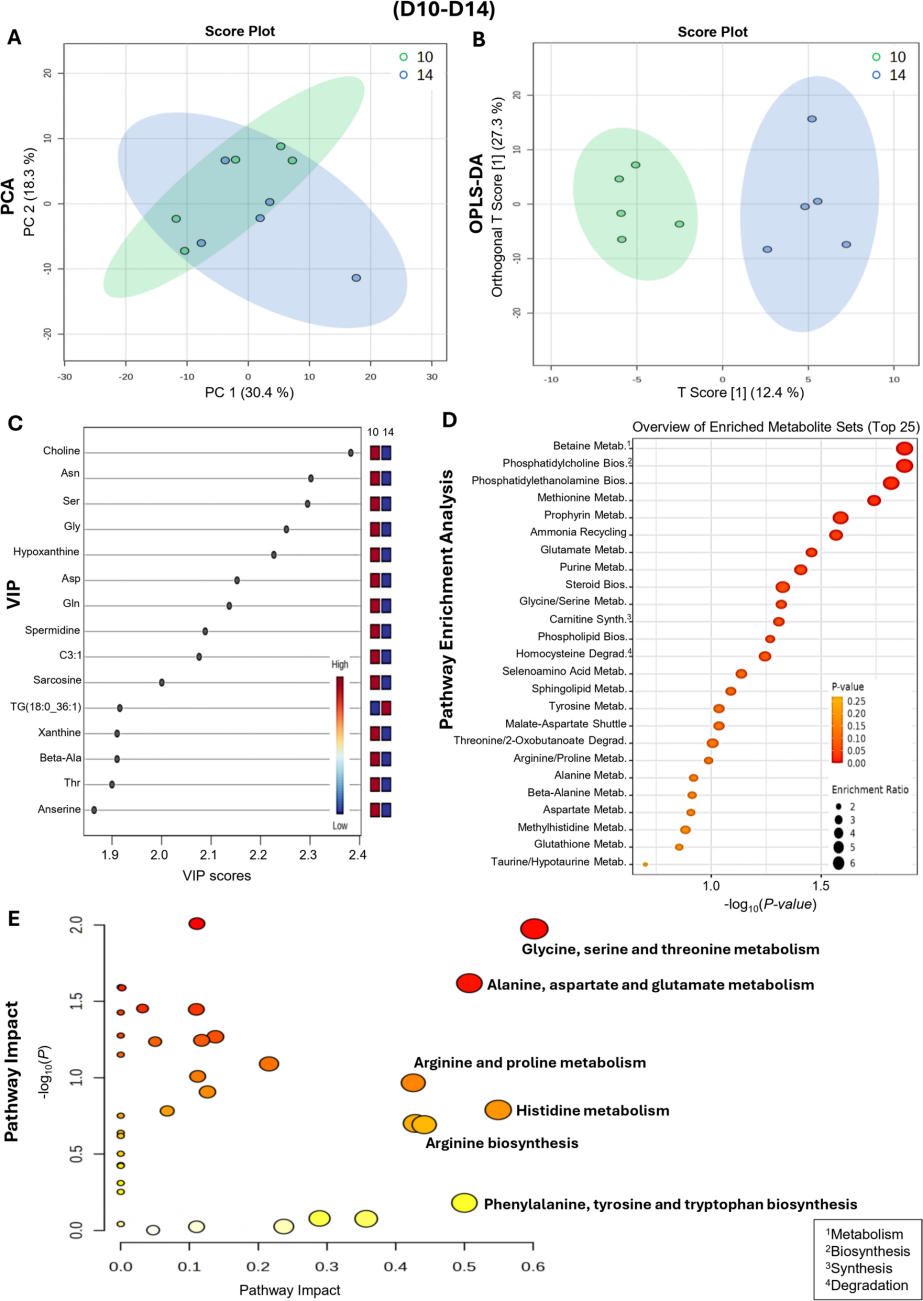
Multivariate and functional enrichment analyses of the uterine luminal metabolome on D10 and D14. **A** Principal component analysis (PCA) score plot of all metabolites for D10–D14. **B** Orthogonal partial least-squares discriminant analysis (OPLS-DA) for D10–D14. **C** Top 15 variable importance in projection (VIP) plot for D10–D14. **D** Quantitative Enrichment Analysis highlighting the metabolic pathways that were enriched in D10–D14. The dots are colored based on the pathway *P*-value and relative size is based on the enrichment ratio. **E** Pathway impact analysis of differentially abundant pathways for D10–D14. Each dot represents an enriched metabolic pathway; dots are colored based on their *P*-value and relative size is based on the pathway impact value

Multivariate and functional enrichment analyses for the main effect of time were conducted by MetaboAnalyst 6.0. Multivariate analyses included principal component analysis (PCA) and partial least squares discriminant analysis (PLS-DA). The PLS-DA analyzes generated ranked lists of variables important for projection (VIPs), which were metabolites that mostly explained the clustering of individuals across time points (i.e., D7, D10 and D14) (Figs. 2, 3 and 4).

### Metabolomics: univariate analysis

Data were analyzed on IBM SPSS Version 29 (IBM Corp., Armonk, NY, USA). A one-way ANOVA was conducted to evaluate the main effect of time on the concentration of metabolites. For multiple comparisons, Tukey’s post-hoc test was applied. The main effect of time was considered significant when *P* < 0.05 and a tendency was considered when 0.05 ≤ *P* ≤ 0.1 (see Additional file 1; Table S1).

## Results

### Metabolomics: data generation and curation

Targeted metabolomics from cytobrush collections enabled the identification and quantification of 630 metabolites; however, after preprocessing the data, 267 metabolites passed the cut-off criteria to be considered for analysis across all three time points.

### Metabolomics: multivariate analysis

Principal component analysis (PCA), including all metabolites that passed the cut-off criteria, revealed an overall similarity among all time points, as depicted in the score plots (Figs. 2A, 3 and 4A). Group sorting was detected when the supervised methods were applied (PLS-DA for D7 to D14 analysis; Fig. 2B, orthogonal partial least squares-discriminant analysis; OPLS-DA for D7–D10; Fig. 3B, and D10–D14 analysis; Fig. 4B). The top 15 greatest VIPs are in Figs. 2C, 3 and 4C. These VIP variables were the most influential on the separation of days. The top 15 VIPs for days separation from D7 to D14 included PC aa C28:1, glutamine, ornithine, TG(18:2_36:4), choline, putrescine, Hex3Cer(d18:1/), Cer(d18:2/14:0), kynurenine, DG(18:1_18:4), PC ae C38:5, HexCer(d18:1/1), PC ae C36:5, arginine, and PC aa C36:4. The top 15 VIPs for days separation from D7 to D10 included PC aa C28:1, putrescine, HexCer(d18:1/1), CE(17:1), TG(18:1_33:1), PC aa C34:3, TG(18:2_36:4), glutamine, PC aa C34:4, ornithine, DG(18:1_18:4), beta-alanine, sarcosine, kynurenine, and Cer(d18:2/14:0). The top 15 VIPs for days separation from D10 to D14 included choline, sarcosine, GABA, serine, glycine, beta-alanine, hypoxanthine, glutamine, TG(14:0_34:2), asparagine, TG(18:0_36:1), aspartate, C3:1, CE(18:0), and PC aa C42:4. Metabolite enrichment analysis revealed overrepresented functional categories mainly related to amino acid metabolism and lipid biosynthesis including, for example, methionine metabolism, glycine and serine metabolism, glutathione metabolism, beta-alanine metabolism, glutamate metabolism, aspartate metabolism, histidine metabolism, alanine metabolism, arginine and proline metabolism, phosphatidylcholine biosynthesis, phosphatidylethanolamine biosynthesis, and sphingolipid metabolism (Figs. 2D, 3 and 4D). Additionally, pathway analysis highlighted arginine and proline metabolism, glycine, serine and threonine metabolism and alanine, aspartate and glutamate metabolism as impacted pathways across time (Figs. 2E, 3 and 4E).

### Metabolomics: univariate analysis

The average concentrations of all 267 detected metabolites on all three time points are provided in Additional file 1 (Table S1) and the changes across time points in all 148 calculated variables are provided in Additional file 1 (Table S2). Table 1 shows the summary of the detected metabolites, grouped by biochemical class, according to their temporal pattern of change. Overall, from the 87 metabolites whose concentrations were affected by day of the estrous cycle, 74 (85%) and 51 (58.6%) increased between D7 and D10 and between D10 and D14, respectively, while 13 (14.9%) and 36 (41.4%) decreased during the same time windows. Thus, there was an overall trend of accumulation of metabolites in the lumen over time, and it was more intense between D7 and D10.

**Table 1:** Number of metabolites detected in uterine luminal fluid and whose concentrations were affected by time, according to biochemical class

Biochemical class	Number of metabolites detected	Number of metabolites affected by time^1^	Direction of change (D7 to D10)		Direction of change (D10 to D14)	
			**Increased**^**2**^	**Decreased**^**3**^	**Increased**^**4**^	**Decreased**^**5**^
Amino acids	17	11	10	1	1	10
Amino acids related	19	4	3	1	1	3
Acylcarnitines	9	2	2	0	0	2
Bile acid	6	2	2	0	0	2
Biogenic amines	6	2	0	2	0	2
Carboxylic acids	4	1	1	0	0	1
Ceramides	21	1	1	0	0	1
Cholesterol esters	20	5	5	0	5	0
Cresols	1	1	1	0	0	1
Diacylglycerols	25	3	1	2	1	2
Dihydroceramides	5	0	0	0	0	0
Fatty acids	2	0	0	0	0	0
Glycerophospholipids	60	33	32	1	31	2
Glycosylceramides	32	5	0	5	5	0
Nucleobases related	2	1	1	0	0	1
Sphingolipids	13	6	6	0	2	4
Triacylglycerols	24	9	8	1	7	2
Vit & Co-Factors	1	1	1	0	0	1
Total	267	87	74	13	51	36

^1^Number of metabolites whose concentrations were affected by time (Significant *P* < 0.05, tendency 0.05 ≤ *P* ≤ 0.1)

^2^Number of metabolites whose concentrations were increased from D7 to D10

^3^Number of metabolites whose concentrations were decreased from D7 to D10

^4^Number of metabolites whose concentrations were increased from D10 to D14

^5^Number of metabolites whose concentrations were decreased from D10 to D14

**Fig. 5:**
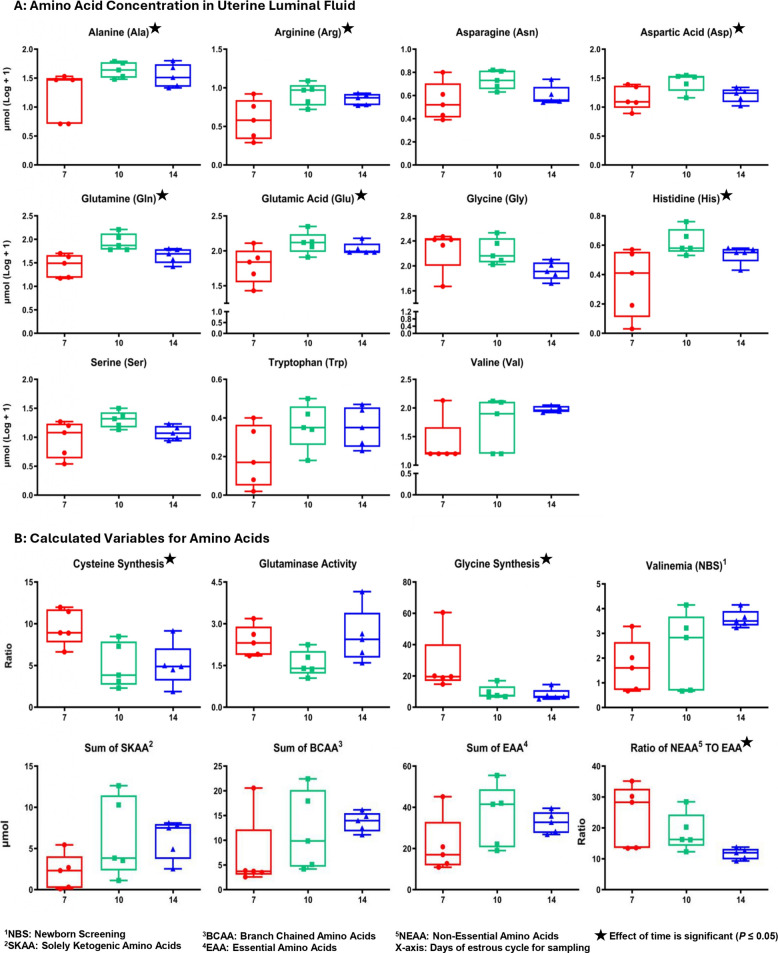
Box-plots of the luminal concentration of amino acids [microMol; Log (x + 1), (**A**)] and calculated variables for amino acids (**B**) affected by time on D7, D10 and D14. The box represents the 25^th^–75^th^ percentile and whiskers minimum and maximum values. Variables were significantly affected by time (*P* < 0.05) or showed a tendency for time effect (0.05 ≤ *P* ≤ 0.1)

**Fig. 6:**
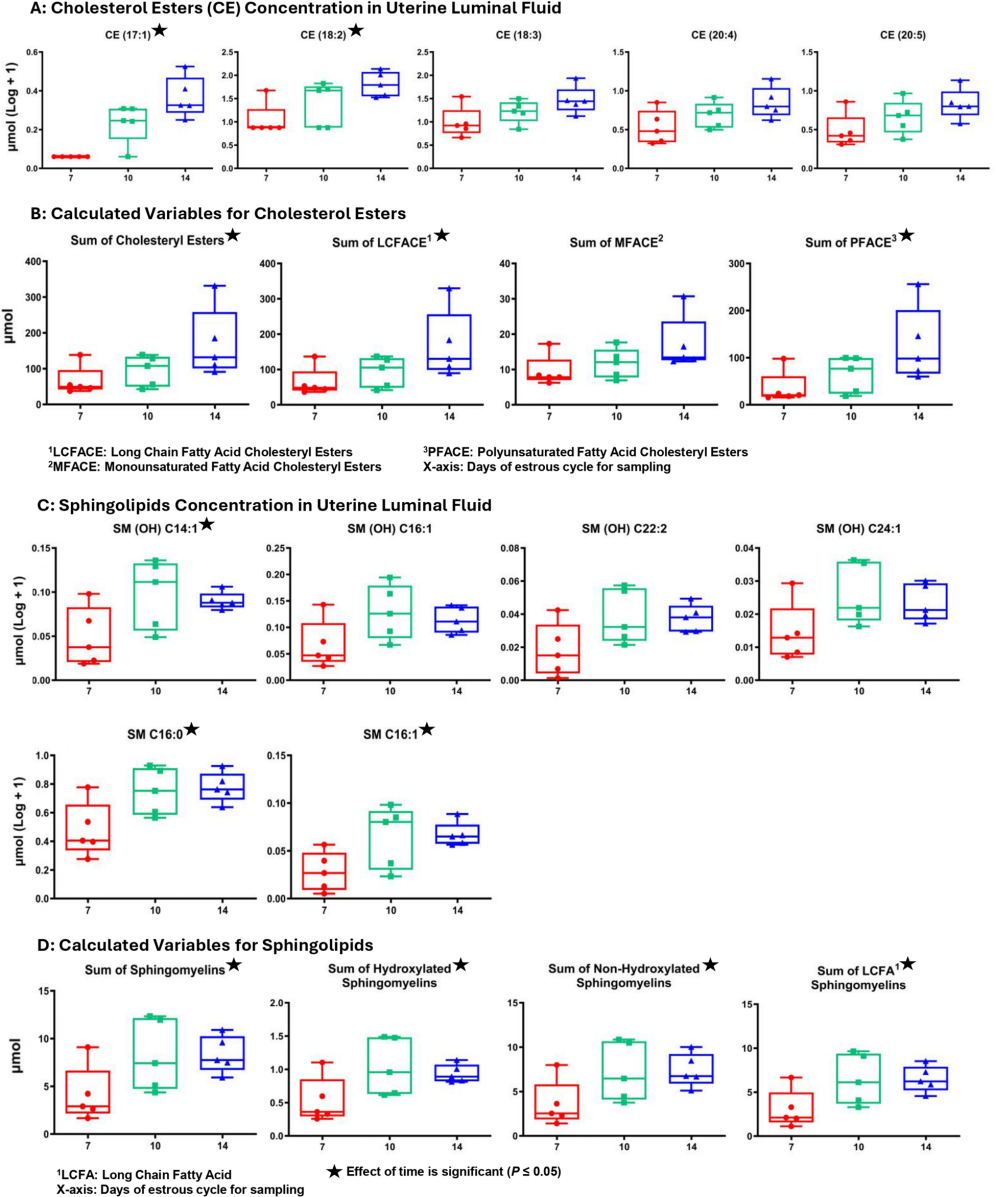
Box-plots of the luminal concentration of cholesterol esters [microMol; Log (*x* + 1), (**A**)] and sphingolipids (**C**), and calculated variables for cholesterol esters (**B**) and sphingolipids (**D**) affected by time on D7, D10 and D14. The box represents the 25^th^–75^th^ percentile and whiskers minimum and maximum values. Variables were significantly affected by time (*P* < 0.05) or showed a tendency for time effect (0.05 ≤ *P* ≤ 0.1)

**Fig. 7:**
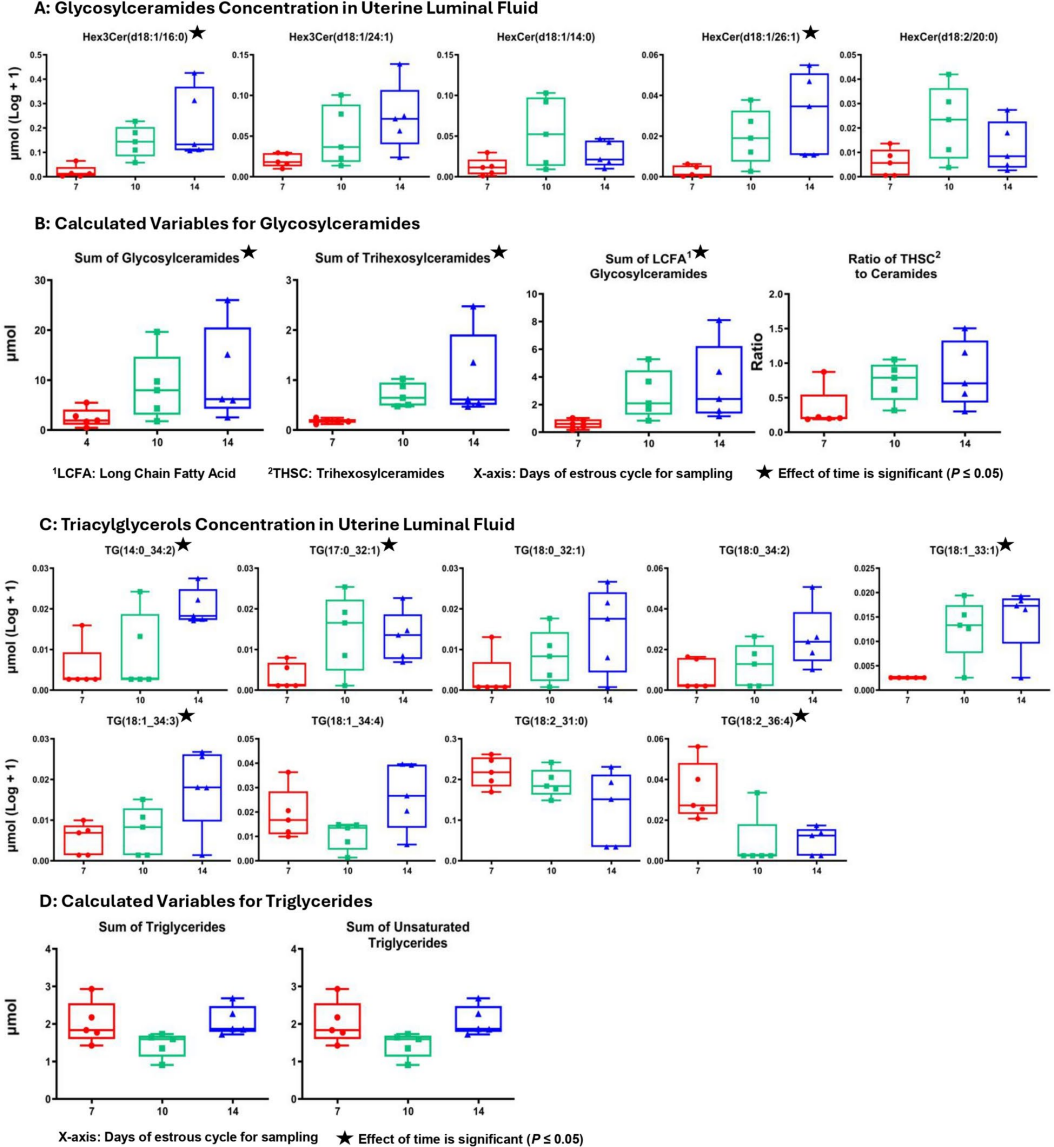
Box-plots of the luminal concentration of glycosylceramides [microMol; Log (*x* + 1), (**A**)] and triacylglycerols (**C**), and calculated variables for glycosylceramides (**B**) and triacylglycerols (**D**) affected by time on D7, D10 and D14. The box represents the 25^th^–75^th^ percentile and whiskers minimum and maximum values. Variables were significantly affected by time (*P* < 0.05) or showed a tendency for time effect (0.05 ≤ *P* ≤ 0.1)

**Fig. 8:**
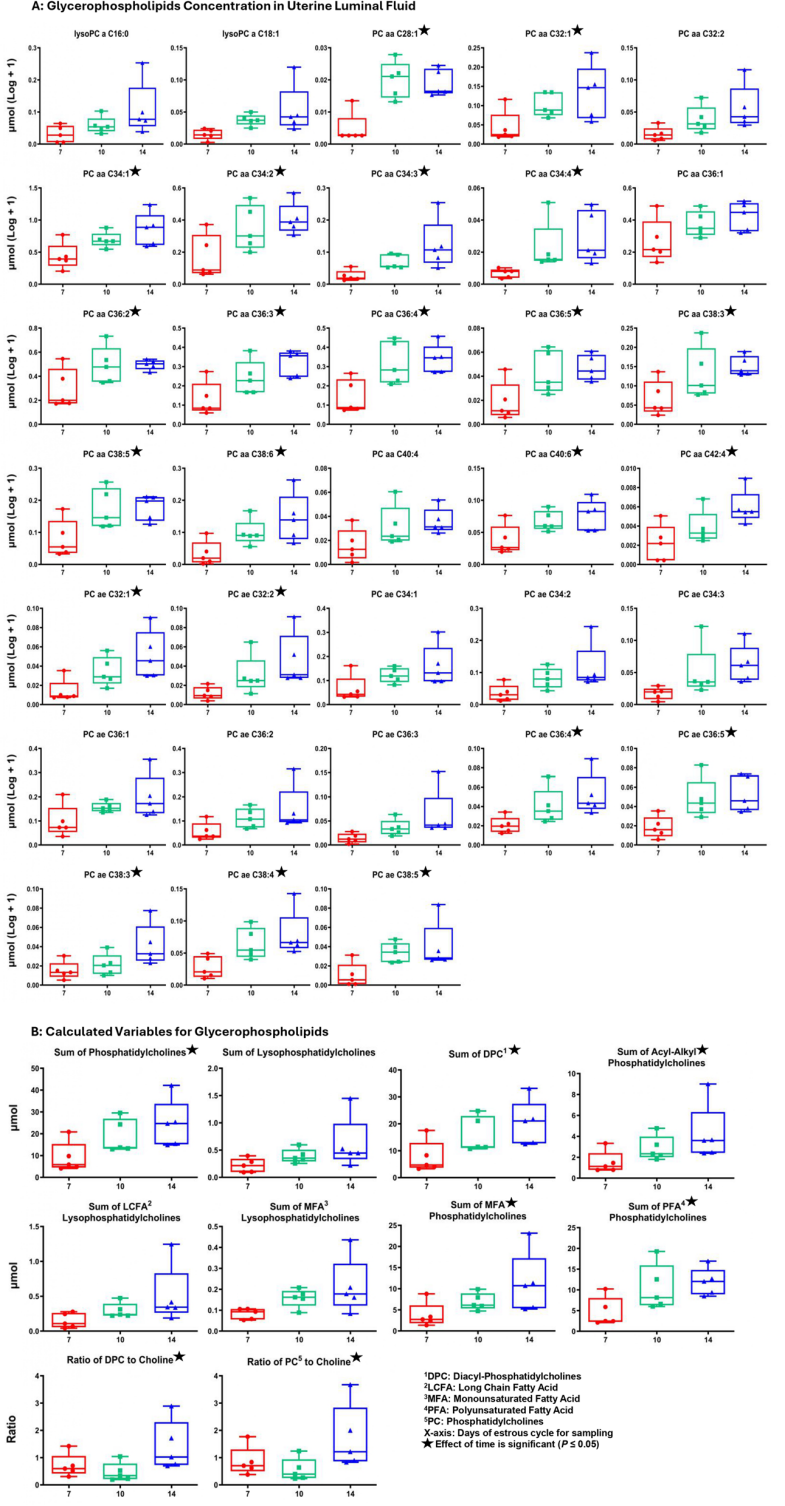
Box-plots of the luminal concentration of glycerophospholipids [microMol; Log (*x* + 1), (**A**)] and calculated variables for glycerophospholipids (**B**) affected by time on D7, D10 and D14. The box represents the 25^th^–75^th^ percentile and whiskers minimum and maximum values. Variables were significantly affected by time (*P* < 0.05) or showed a tendency for time effect (0.05 ≤ *P* ≤ 0.1)

It was remarkable that amino acids, amino acids related and sphingolipid metabolites showed a general decrease between D10 and D14. Specifically, the concentration of three essential (arginine, glutamine, and histidine) and three non-essential (alanine, aspartic acid, and glutamic acid) amino acids changed over time significantly (*P* < 0.05), while the concentration of two essential (tryptophan and valine) and three non-essential (asparagine, glycine and serine) amino acids showed a tendency to change (0.05 ≤ *P* ≤ 0.1), out of the 17 amino acids detected in the uterine fluid. In contrast, the concentration of two cholesterol esters, three sphingolipids, two glycosylceramides, five triacylglycerols, and 22 glycerophospholipids changed overtime significantly (*P* < 0.05), while the concentration of three cholesterol esters, three sphingolipids, three glycosylceramides, four triacylglycerols, and 11 glycerophospholipids showed a tendency to change (0.05 ≤ *P* ≤ 0.1) out of 20, 13, 32, 24 and 60 detected in the uterine fluid, respectively. In general, there was a continuous accumulation of lipids in the uterine fluid from D7 to D14. Time changes in the concentrations of individual amino acids, lipids and their calculated variables are in Figs. 5, 6, 7 and 8. The concentrations of alanine, arginine, aspartic acid, glutamine, glutamic acid and histidine significantly increased, while the concentrations of asparagine, serine, and tryptophan tended to increase from D7 to D10 and decreased from D10 to D14 (Fig. 5A). For calculated variables, the concentration of essential amino acids tended to increase between D7 and D10, and ratio of non-essential to essential amino acids decreased between D7 to D14 (Fig. 5B).

The concentrations of cholesterol esters [CE (17:1) and CE (18:2)] significantly increased, and the concentrations of CE (18:3), CE (20:4), and CE (20:5) tended to increase from D7 to D14 (Fig. 6A), while for the calculated variables of cholesterol esters, the sum of long chain fatty acid cholesterol esters and sum of polyunsaturated fatty acid cholesterol esters significantly increased from D7 to D14 (Fig. 6B). The concentrations of sphingolipids (SM(OH)C14:1, SM C16:1) significantly increased from D7 to D10, while the concentration of SM C16:0, and sum of sphingomyelins, sum of non-hydroxylated sphingomyelins, and sum of long chain fatty acid sphingomyelins significantly increased from D7 to D14 (Fig. 6C and D). The concentrations of Hex3Cer(d18:1/16:0), HexCer(d18:1/26:1), sum of trihexosylceramides, and sum of long chain glycosylceramides significantly increased from D7 to D14, while Hex3Cer(d18:1/24:1) showed the tendency to increase (Fig. 7A and B). The concentrations of triacylglycerols TG(14:0_34:2), TG(18:1_33:1), and TG(18:1_34:3) significantly increased from D7 to D14, and the concentrations of TG(18:0_32:1), and TG(18:0_34:2) showed a tendency of increase (Fig. 7C). The sum of triglycerides and sum of unsaturated triglycerides showed a tendency to decrease from D7 to D10 and then increase to D14 (Fig. 7D).

Among 22 glycerophospholipids, the concentrations significantly increased from D7 to D14 except for phosphatidylcholine C28:1, C36:2 and C38:5 (Fig. 8A), and 11 additional glycerophospholipids showed a tendency to increase from D7 to D14. For the calculated variables, the sum of phosphatidylcholines, sum of diacyl-phosphatidylcholines, sum of acyl-alkyl phosphatidylcholines, sum of monounsaturated fatty acid phosphatidylcholines, and sum of polyunsaturated fatty acid phosphatidylcholines significantly increased from D7 to D14, while the ratios of diacyl-phosphatidylcholine to choline and phosphatidylcholine to choline significantly decreased from D7 to D10 and then increased to D14 (Fig. 8B).

### RNA sequencing: data analysis

**Fig. 9:**
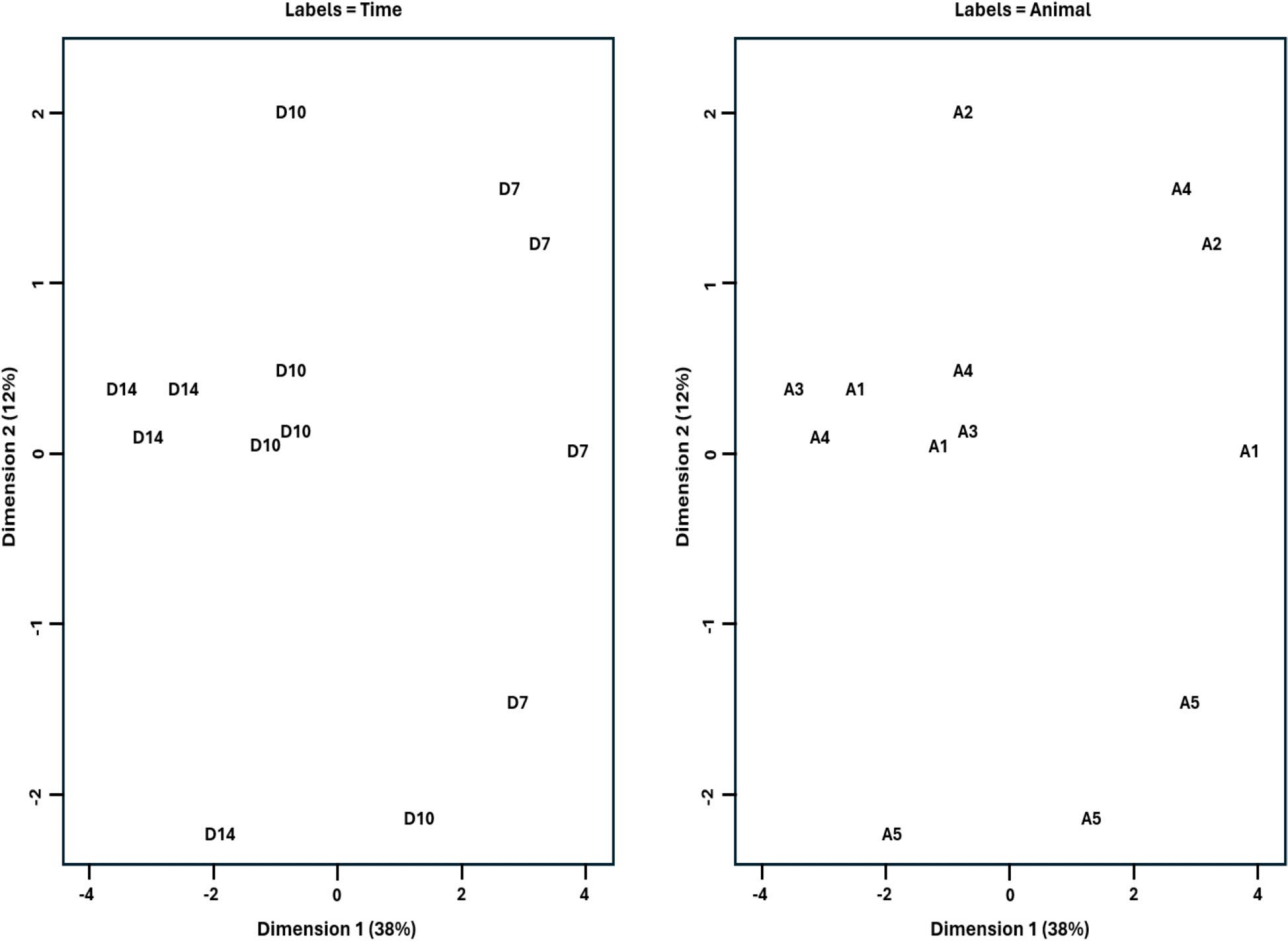
Multidimensional Scaling Plot of endometrial transcriptomic profiles. The samples are plotted in two-dimensional space. Left panel shows samples labelled by sampling days (D7, D10, D14). Right panel shows samples labelled by individual animal (A1–A5)

The multidimensional scaling (MDS) plot reveals the relative similarities among the samples analyzed. Dimension 1 distinctly separates the samples according to sampling day, while Dimension 2 differentiates them by individual animal. This pattern indicates that biological replicates are consistent within sampling day, which differ among them, reflecting the temporal effect in global gene expression (Fig. 9).

### Gene expression patterns: overrepresentation analysis

Eight different patterns of gene expression across D7, D10, and D14 were identified (A–H, see Additional file 3; Fig. S3). The details of genes in each pattern are given in Additional file 1 (Tables S3–S10). These lists were regrouped according to increased or decreased temporal patterns between D7–D10 and D10–D14 and four new lists (I–L) of genes were obtained (pattern I: genes that increased expression between D7 and D10, pattern J: genes that decreased expression between D7 and D10, pattern K: genes that increased expression between D10 and D14, and pattern L: genes that decreased expression between D10 and D14. The details of genes in patterns I to L are given in Additional file 1 (Tables S11–S14). The lists of genes in patterns I to L were analyzed through the EnrichKit database, and the top 50 pathways (FDR ≤ 0.1) were selected.

When considering genes that showed expression that increased or tended to increase between D7 and D10, there was an enrichment in the following pathways: bile secretion, oxidative phosphorylation, calcium ion binding, plasma membrane activity, extracellular secretion, peptidase and metallopeptidase activity, and cytokine–cytokine receptor interaction, reflecting enhanced digestion, energy metabolism, protein turnover, transport, and signaling functions. During the same period (between D7 and D10), arginine and proline metabolism, histidine metabolism, general metabolic pathways, triglyceride lipase activity, vitamin digestion and absorption, sirtuin regulation, and SLC-mediated transport were decreased. These patterns are consistent with reduced amino acid metabolism, reduced lipid mobilization, and reduced nutrient transport. Overall, between D7 and D10, uterine function prioritized increased secretory, proliferative, energetic, and signaling capacity while reducing intracellular amino acid and lipid utilization and uptake, favoring metabolite accumulation in the uterine lumen. From D10 to D14, the pathways that were enriched, based on genes that were upregulated or tended to be upregulated in that window, included fatty acid elongation, sphingolipid metabolism, carbon and 2-oxocarboxylic acid metabolism, ABC and SLC transport, and IGF transport and uptake, suggesting an increased lipid synthesis, increased amino acid metabolism, and increased nutrient signaling. In contrast, genes whose expression decreased or tended to decrease between D10 and D14 resulted in pathways including protein digestion and absorption, bile acid metabolism, insulin secretion (including regulation by free fatty acids and GPR40), nuclear receptor transcription pathways, and PI3K/AKT signaling, pointing to a decline in nutrient absorption, decreased lipid-derived regulation, and reduced integrated metabolic signaling. Collectively, between D10 and D14 the uterus intensified continuous secretions, lipid synthesis, increased amino acid utilization for energy and protein synthesis, and enhanced membrane transport capacity, while reducing extracellular proteolytic activity and metabolic/hormonal integration, favoring in the preparation for the potential pregnancy in case of the presence of embryo. For both periods the data is provided in Additional file 1 (Tables S15–S18).

### Targeted analysis of enriched pathways

To determine the relevance of the enriched pathways with amino acid and lipid metabolism as well as nutrient transport, the list of pathways obtained with genes that increased expression between D7 and D10, decreased expression between D7 and D10, increased expression between D10 and D14 and decreased expression between D10 and D14 (see Additional file 1; Tables S11–S14) were each further searched for the following keywords: amino acids, alanine, arginine, asparagine, aspartic acid, glutamine, glutamic acid, glycine, histidine, serine, tryptophan, valine, lipids, ceramides, cholesterol, fatty acid, sphingolipids and transport. The number of pathways containing at least one of these keywords was then tabulated for each temporal contrast and expression direction. Overall, the keywords related search identified 69 pathways (i.e., Cellular amino acid biosynthetic process, Metabolism of amino acids and derivatives, Alanine, aspartate and glutamate metabolism, Lipid metabolic process, Ceramide biosynthetic process, Fatty acid biosynthesis, Transmembrane transporter activity) from the list of genes that increased or tended to increase expression between D7 to D10 and 79 pathways (i.e., Arginine and proline metabolism, Glyoxylate metabolism and glycine degradation, Transmembrane transporter activity, Transport of inorganic cations/anions and amino acids/oligopeptides, Lipid metabolic process, Transport of small molecules) from D10 to D14 (Tables 2 and 3, respectively).

**Table 2:** Pathways generated from functional enrichment analysis of transcripts whose expression increased or tended to increase between D7 and D10, curated according to specific keywords

Keywords^1^	Number of pathways^2^	Pathways^3^	Representative genes^4^*
Amino acids	8	Cellular amino acid biosynthetic process; Aromatic amino acid family metabolic process; Amino acid permease/SLC12A domain; Amino Acid Sequence; Sequence Homology, Amino Acid; Amino Acid Substitution; Amino Acids; Metabolism of amino acids and derivatives	***ALDH4A1****, ****ASS1****, ****DAO****, ****GLUL****,**** GOT1****,**** HPD, SLC1A3****, ****SLC2A1, SLC12A1, SLC12A2****, ****TAT,**** AASS, BCKDK, ENOPH1, FAH, GATM, MRI1, MTR, PSPH, PYCR1, SLC25A15*
Alanine	2	Alanine, aspartate and glutamate metabolism—*Bos taurus* (cow); Phenylalanine metabolism—*Bos taurus* (cow)	***ALDH4A1, ALDH5A1****, ****ASS1, GAD1****, ****GLS****, ****GLUL, GOT1, TAT,**** DDC, HPD*
Arginine	2	Arginine biosynthesis—*Bos taurus* (cow); Arginine	*ASS1, GLP1R, GLS, GLUL, GOT1, MSTN, NOS3*
Asparagine	0	N/A	N/A
Aspartic acid	4	Aspartic-type endopeptidase activity; Aspartic peptidase A1 family; Aspartic peptidase, active site; Aspartic Acid Endopeptidases	***CCN1, CTSS, ECE1, EDNRB, NOS3,**** BACE2, CASP3, PSEN1*
Glutamine	0	N/A	N/A
Glutamic acid	1	Glutamic Acid	*A2M, ****ALDH2****, CYP11A1, SLC1A3, SLC2A1*
Glycine	0	N/A	N/A
Histidine	0	N/A	N/A
Serine	6	Serine-type endopeptidase inhibitor activity; Serine-type endopeptidase activity; serine C-palmitoyltransferase activity; Regulation of cyclin-dependent protein serine/threonine kinase activity; Serine-type peptidase activity; Negative regulation of protein serine/threonine kinase activity	***DPP4, HTRA1, PAPLN, PCSK6, PRSS22, PRSS33, PRSS50, RHBDD2, RHBDL2, SEC11A, SEC11C, SERPINB1, SERPINB5, SERPINB7, SERPINB8, SERPINB9, SERPINB12, SPTLC2, SPTSSA, SPTSSB, TMPRSS4, TMPRSS6, TMPRSS11D, TMPRSS11E,**** PLAT, PLAU, PRSS8*
Tryptophan	0	N/A	N/A
Valine	0	N/A	N/A
Lipids	10	Lipid metabolic process; Lipid oxidation; ATPase-coupled intramembrane lipid transporter activity; Positive regulation of lipid storage; Lipid translocation; Lipid catabolic process; Lysophospholipids; Phospholipids; Lipid Metabolism; Lipid Bilayers	***AACS, ACACA, ACSF3, ACSL6, ACSS1, ALOX12, ALOX12B, ALOX15B, ATP8A2, ATP8B1, ATP9A, ATP11B, CPT2, CYP1A1, CYP2C87, CYP11A1, DEGS1, DGKE, ELOVL2, FA2H, FABP5, FADS2B, FADS3, GALC, GDPD2, GPCPD1, GPLD1, HADHA, HMGCS1, LCAT, NPC2, NR1H2, PCTP, PLA2G4A, PLA2G4F, PLCD1, PLCE1, PLCH2, PLIN3, PNPLA1, SCD5, VLDLR***
Ceramides	1	Ceramide biosynthetic process	***AGK, ALOX12B, FA2H, PNPLA1, SPTLC2, SPTSSA, SPTSSB***
Cholesterol	2	Cholesterol homeostasis; Cholesterol	***ABCB11, CYP11A1, CYP51A1, LCAT, LDLRAP1, NPC2, NR1H2, PON1, PON3, SOAT1, SREBF2, VLDLR***
Fatty acids	12	Long-chain fatty acid transporter activity; Fatty acid transmembrane transporter activity; Fatty acid metabolic process; Long-chain fatty acid metabolic process; Fatty acid transport; Long-chain fatty acid transport; Unsaturated fatty acid biosynthetic process; Fatty acid desaturase domain; Fatty acid metabolism—*Bos taurus* (cow); Fatty acid degradation—*Bos taurus* (cow); Fatty acid biosynthesis—*Bos taurus* (cow); Fatty acid metabolism	***AACS, ABCB11, ACACA, ACADVL, ACAT1, ACAT2, ACSF2, ACSF3, ACSL5, ACSL6, ALOX12, ALOX12B, CPT2, CYP1A1, DEGS1, ELOVL2, FA2H, FABP5, FADS2B, FADS3, GPX1, HADHA, HADHB, MECR, MFSD2A, MID1IP1, PCTP, PLA2G4A, PON1, PON3, SCD5, SGPL1, SLC27A1, SLC27A2, SLC27A5***
Sphingolipids	0	N/A	N/A
Transporter	21	Long-chain fatty acid transporter activity; Bile acid and bile salt transport; Transmembrane transporter activity; Fatty acid transmembrane transporter activity; Transporter activity; Leukotriene transport; Fatty acid transport; Transmembrane transport; ABC-type transporter activity; ATPase-coupled intramembrane lipid transporter activity; Long-chain fatty acid transport; Transmembrane transporter binding; ABC transporter type 1, transmembrane domain; ABC transporter type 1, transmembrane domain superfamily; SLC12A transporter, C-terminal; SLC12A transporter family; ABC transporter-like, conserved site; ABC transporter-like; ABC transporters—*Bos taurus* (cow); ATP-Binding Cassette Transporters; Transport of small molecules	***ABCB9, ABCC5, ACACA, ACADVL, ACAT1, ACAT2, ACSL5, ACSL6, ACSF3, ADH6, ALDH2, ALOX12B, ATP2A1, ATP2A3, ATP2B4, ATP8A2, ATP8B1, ATP12A, ATP13A4, CPT2, CYP1A1, ELOVL2, FABP5, GPX1, HADHA, HADHB, LCAT, MECR, MFSD2A, NOS3, NPC2, NR1H2, PLA2G4A, RHBG, SCD5, SLC1A3, SLC2A1, SLC2A3, SLC4A3, SLC5A8, SLC9A1, SLC12A4, SLC13A2, SLC16A1, SLC22A15, SLC22A16, SLC22A17, SLC26A7, SLC26A9, SLC27A1, SLC27A2, SLC27A5, SLC29A1, SLC36A2, SLC39A2, SLC46A1, SLCO3A1, SOAT1, SPNS2, SREBF2***
Total	69	–	–

^*^Representative genes: Bold font = *P* < 0.05, Not bold = 0.05 ≤ *P* ≤ 0.1

^1^Keywords used to probe the list of pathways obtained from the lists generated with genes whose expression increased between D7 and D10

^2^The number of pathways containing at least one of these keywords

^3^Name of all obtained pathways for each selected keyword

^4^Relative genes from obtained pathways containing at least one of these keywords

**Table 3:** Pathways generated from functional enrichment analysis of transcripts whose expression increased or tended to increase between D10 and D14, curated according to specific keywords

Keywords^1^		Number of pathways^2^	Pathways^3^		Representative genes^4^*
Amino acids		8	Biosynthesis of amino acids—*Bos taurus* (cow); Amino sugar and nucleotide sugar metabolism—*Bos taurus* (cow); Amino Acid Sequence; Sequence Homology, Amino Acid; Amino Acids; Amino Acid Isomerases; Amino Acid Substitution; Transport of inorganic cations/anions and amino acids/oligopeptides		***ADA, ALDH1A1, ALDH2, ARG1, ASL, ASS1, CAD, CKB, CPS1, DGAT1, FASN, FABP3, GLUD1, GOT2, GPT2, HADHA, HSD17B10, MFGE8, NAALAD2, ODC1, P4HB, PHGDH, PRODH, RENBP, SAT1, SLC2A1, SLC3A2, SLC6A11, SLC6A20, SLC7A4, SLC7A8, SLC12A2, SLC15A4, SLC16A1, SLC25A10, SLC25A22, SLC25A29, SLC26A7, SLC36A2, SLC43A2, SMOX, SMS, TCN2, TSFM, TUFM***
Alanine		2	beta-Alanine metabolism—*Bos taurus* (cow); Alanine		***ALDH2, CNDP1***
Arginine		3	Arginine biosynthesis—*Bos taurus* (cow); Arginine and proline metabolism—*Bos taurus* (cow); Arginine		***ARG1, ASL, ASS1, GATM, GOT2, GPT2, PADI2***
Asparagine		2	Cysteine peptidase, asparagine active site; Asparagine		***ADA***
Aspartic acid		0	N/A		N/A
Glutamine		0	N/A		N/A
Glutamic acid		1	Glutamic Acid		***ALDH2, PGK1, SLC2A1***
Glycine		2	Glycine; Glyoxylate metabolism and glycine degradation		***DAO, GOT2***
Histidine		0	N/A		N/A
Serine		5	Serine-type endopeptidase inhibitor activity; Protein serine/threonine kinase activity; Serine/threonine-protein kinase, active site; Serine proteases, trypsin family, serine active site; Serine proteases, trypsin family, histidine active site		***CAMK2G, F2, GZMA, KLK12, MAP2K3, MAP2K6, MAST2, PLG, PRSS8, RIPK3, RPS6KA4***
Tryptophan		0	N/A		N/A
Valine		0	N/A		N/A
Lipids		16	Lipid translocation; Sphingolipid biosynthetic process; Cellular lipid metabolic process; ATPase-coupled intramembrane lipid transporter activity; Phospholipid-translocating ATPase complex; Phospholipid transport; Lipid metabolic process; Lipid biosynthetic process; Phospholipid metabolic process; Intracellular lipid binding protein; Sphingolipid metabolism—*Bos taurus* (cow); Ether lipid metabolism—*Bos taurus* (cow); Glycosphingolipid biosynthesis—globo and isoglobo series—*Bos taurus* (cow); Glycerolipid metabolism—*Bos taurus* (cow); Lipid metabolism; Sphingolipid metabolism		***ABCA3, ACAD8, ACER1, AGPAT5, ANGPTL8, ARSL, ATP9A, ATP10A, ATP10B, ATP11A, ATP11B, CERS4, CRABP1, CRABP2, DGAT1, DGKQ, DEGS1, ELOVL2, ELOVL6, FADS3, FABP3, FASN, GDPD3, HADHA, HEXB, LIPH, NAGA, NEU1, PAFAH2, PLA2G4F, PLB1, PLD1, PLPP1, PLPP3, PITPNM1, PTDSS2, PTGES2, RBP1, SPTLC2, UQCRC1, VLDLR***
Ceramides		1	Ceramide metabolic process		***ACER1****, ****PLPP3****, ****PLPP1***
Cholesterol		0	N/A		N/A
Fatty acids		15	Fatty acid elongase activity; Fatty acid elongation, saturated fatty acid; Unsaturated fatty acid biosynthetic process; Fatty acid transport; Fatty acid transmembrane transporter activity; Fatty acid elongation, polyunsaturated fatty acid; Long-chain fatty acid transporter activity; Very long-chain fatty acid biosynthetic process; Fatty acid biosynthetic process; Very long-chain fatty acid metabolic process; Fatty acid metabolic process; Long-chain fatty acid metabolic process; Fatty acid elongation—*Bos taurus* (cow); Fatty Acids, Unsaturated; Fatty acid metabolism		***ACAD9, ACOT8, ACSF2, CYP1A1, CYP1A2, DECR2, DGAT1, ELOVL2, ELOVL4, ELOVL6, FASN, FABP3, HADHA, MFSD2A, PON2, PPT1, PTGES2, SLC27A2, SLC27A5***
Sphingolipids		0	N/A		N/A
Transporter		24	Transmembrane transporter activity; Transmembrane transport; ABC-type transporter activity; Transporter activity; Fatty acid transport; Fatty acid transmembrane transporter activity; Long-chain fatty acid transporter activity; ATPase-coupled intramembrane lipid transporter activity; Phospholipid transport; Efflux transmembrane transporter activity; Protein transmembrane transporter activity; Xenobiotic transport; ATPase-coupled transmembrane transporter activity; ABC transporter-like; ABC transporter-like, conserved site; MFS transporter superfamily; ABC transporter type 1, transmembrane domain; ABC transporter type 1, transmembrane domain superfamily; SLC26A/SulP transporter; SLC26A/SulP transporter domain; ABC transporters—*Bos taurus* (cow); Transport of small molecules; SLC-mediated transmembrane transport; Transport of inorganic cations/anions and amino acids/oligopeptides		***ABCA3, ABCA5, ABCB1, ABCB5, ABCB6, ABCC3, ABCC4, ABCC10, ABCE1ABCF2, AFG3L2, ANGPTL8, ASIC2, ATP2B2, ATP6V0D2, ATP7B, ATP9A, ATP10A, ATP10B, ATP11A, ATP13A4, CACNA1E, CLCN5, CUBN, CYB5R1, ERLIN1, GJB2, GJB6, HCN2, LC35F2, LRRC8D, MFSD2A, MFSD10, MRS2, PHB1, PHB2, PITPNM1, RIPK3, SCNN1B, SCNN1G, SFXN2, SLC2A1, SLC2A5, SLC4A4, SLC5A5, SLC5A11, SLC7A8, SLC12A1, SLC12A2, SLC13A2, SLC15A1, SLC15A4, SLC16A1, SLC16A3, SLC16A12, SLC20A1, SLC23A3, SLC25A22, SLC26A1, SLC26A4, SLC26A7, SLC27A2, SLC27A5, SLC29A1, SLC39A2, SLC39A11, SLC43A2, SLC44A2, SLC44A5, SLC45A2, SLC49A3, SLC35B2, SLC35C1, SLCO2A1, SPNS1, STEAP3, TFRC, TIMM17A, TIMM23, TRPC6, TRPM1, TRPV6, UNC93A***
Total	79		–		*–*

^*^Representative genes: Bold font = *P* < 0.05, Not bold = 0.05 ≤ *P* ≤ 0.1

^1^Keywords used to probe the list of pathways obtained from the lists generated with genes whose expression increased between D10 and D14

^2^The number of pathways containing at least one of these keywords

^3^Name of all obtained pathways for each selected keyword

^4^Relative genes from obtained pathways containing at least one of these keywords

In contrast, 35 pathways (i.e., Metabolism of amino acids and derivatives, Transport of inorganic cations/anions and amino acids/oligopeptides, Amino acid transport, Cholesterol metabolism, Cation transmembrane transport) were detected from genes with expression that decreased or tended to decrease between D7 and D10, and 30 pathways (i.e., Arginine and proline metabolism, Amino acid/polyamine transporter I, Lipid catabolic process, Transmembrane transporter activity) between D10 and D14 (Tables 4 and 5, respectively).

**Table 4:** Pathways generated from functional enrichment analysis of transcripts whose expression decreased or tended to decrease between D7 and D10, curated according to specific keywords

Keywords^1^	Number of pathways^2^	Pathways^3^	Representative genes^4^*
Amino acids	4	Amino acid transport; Biosynthesis of amino acids—*Bos taurus* (cow); Metabolism of amino acids and derivatives; Transport of inorganic cations/anions and amino acids/oligopeptides	***ALDH18A1, ASL, CARNMT1, GCDH, GCSH, GLUD1, HSD17B10, LIPT2, MAT1A, MAT2A, NAALAD2, ODC1, PHGDH, SAT1, SLC3A2, SLC4A9, SLC6A8, SLC6A9, SLC6A11, SLC6A20, SLC7A4, SLC9A2, SLC12A5, SLC24A4, SLC25A10, SLC25A22, SLC25A29, SMOX, SMS***
Alanine	0	N/A	N/A
Arginine	1	Arginine and proline metabolism—*Bos taurus* (cow)	***ALDH18A1, CKB, ODC1, PRODH, SAT1, SMOX, SMS***
Asparagine	0	N/A	N/A
Aspartic acid	0	N/A	N/A
Glutamine	1	Glutamine metabolic process	*CAD, GLUD1*
Glutamic acid	0	N/A	N/A
Glycine	0	N/A	N/A
Histidine	1	Histidine metabolism—*Bos taurus* (cow)	***CARNS1, CARNMT1, CNDP1, CNDP2, HNMT***
Serine	2	Negative regulation of peptidyl-serine phosphorylation; Cyclin-dependent protein serine/threonine kinase inhibitor activity	***EPM2A, MLXIPL***
Tryptophan	0	N/A	N/A
Valine	0	N/A	N/A
Lipids	3	Glycerolipid metabolic process; Glycerolipid metabolism—*Bos taurus* (cow); Lipids	***AKR1B1, DGKA, DGKI, DGKQ, INSR, LIPG, LPL, LPIN1, MBOAT2, PPARGC1A, SREBF1, THRSP***
Ceramides	0	N/A	N/A
Cholesterol	3	Cholesterol metabolic process; Cholesterol efflux; Cholesterol metabolism—*Bos taurus* (cow)	***LIPA, LIPG, SULT2B1,**** ANGPTL8, APOE, LPL, LRP2, SCAP,*
Fatty acids	9	Very long-chain fatty acid-CoA ligase activity; Long-chain fatty acid transport; Long-chain fatty acid metabolic process; Very long-chain fatty acid biosynthetic process; Fatty acid biosynthesis—*Bos taurus* (cow); Fatty acid transport proteins; Fatty acids; Fatty acyl-CoA biosynthesis; Fatty acid metabolism	***ABCC1, ACAD11, ACSL3, ACSL4, CBR4, CIDEA, DECR2, ELOVL7, LPIN1, LPL, HSD17B8, PECR, PPARD, SLC27A3, SLC27A4, SLC27A6,**** SREBF1, TECR, THRSP*
Sphingolipids	0	N/A	N/A
Transporter	11	Cation transport; Cation transmembrane transport; Cation transmembrane transporter activity; Amino acid transport; Long-chain fatty acid transport; L-glutamate transmembrane transport; Fatty acid transport proteins; Active transport, Cell nucleus; SLC-mediated transmembrane transport; Transport of bile salts and organic acids, metal ions and amine compounds; Transport of inorganic cations/anions and amino acids/oligopeptides	***ANO10, ARL2BP, ATP7B, MCOLN2, MCOLN3, SCN5A, SCN11A, SLC3A2, SLC6A9, SLC6A11, SLC6A20, SLC7A4, SLC9A2, SLC9A4, SLC12A5, ATP13A3, SLC13A5, SLC24A4, SLC25A10, SLC25A13, SLC25A22, SLC25A29, SLC27A3, SLC27A4, SLC27A6, SLC30A2, SLC30A4, SLC30A5, SLC30A9, SLC30A10, SLC33A1, SLC39A8, SLC44A2, TMEM63C***
Total	35	–	*–*

^*^Representative genes: Bold font = *P* < 0.05, Not bold = 0.05 ≤ *P* ≤ 0.1

^1^Keywords used to probe the list of pathways obtained from the lists generated with genes whose expression decreased between D7 and D10

^2^The number of pathways containing at least one of these keywords

^3^Name of all obtained pathways for each selected keyword

^4^Relative genes from obtained pathways containing at least one of these keywords

**Table 5:** Pathways generated from functional enrichment analysis of transcripts whose expression decreased or tended to decrease between D10 and D14, curated according to specific keywords

Keywords^1^	Number of pathways^2^	Pathways^3^	Representative genes^4^*
Amino acids	2	Amino acid/polyamine transporter I; Cationic amino acid transporter, C-terminal	***LOC509649, LOC512219, SLC7A4, SLC7A7***
Alanine	0	N/A	N/A
Arginine	1	Arginine and proline metabolism—*Bos taurus* (cow)	*ALDH18A1, CARNS1, PRODH, SAT1, SAT2*
Asparagine	0	N/A	N/A
Aspartic acid	0	N/A	N/A
Glutamine	0	N/A	N/A
Glutamic acid	0	N/A	N/A
Glycine	0	N/A	N/A
Histidine	0	N/A	N/A
Serine	5	Protein tyrosine/serine/threonine phosphatase activity; MAP kinase tyrosine/serine/threonine phosphatase activity; Protein serine/threonine phosphatase activity; Serine/threonine-protein kinase, active site; Serine	***ANXA5, CAMK2D, CDC14A, CDK7, DUSP8, DUSP10, DUSP16, ESR1, ESR2, MAPK8, PLK2, PPM1H, PPM1K, PPP3CA, PPP2CB, PRKCG, PTP4A1, SIK3, ZFP36***
Tryptophan	0	N/A	N/A
Valine	0	N/A	N/A
Lipids	8	Sphingolipid metabolic process; ATPase-coupled intramembrane lipid transporter activity; Lipid catabolic process; Lipid storage; Sphingolipid metabolism—*Bos taurus* (cow); Sphingolipid signaling pathway—*Bos taurus* (cow); Regulation of lipid metabolism by PPARalpha; Sphingolipid metabolism	***ACER2, ATP8B1, ATP8B2, BCL2, CERS6, CIDEA, GALC, GM2A, GNAQ, MAPK8, NCOA1, NCOA2, PLA2G2F, PLCB1, PLCL2, PLIN2, PPARA, PPP2CB, PPP2R5E, PRKD3, PRKCG, SGPP1, SMPD3, SPNS2, ST3GAL2***
Ceramides	0	N/A	N/A
Cholesterol	1	Synthesis of bile acids and bile salts via 7alpha-hydroxycholesterol	***LOC782061, NCOA1, NCOA2***
Fatty acids	3	Long-chain fatty acid transport; Negative regulation of fatty acid oxidation; Long-chain fatty acid transporter activity	***ABCD1, FABP5, SLC27A1, PLIN2***
Sphingolipids	0	N/A	N/A
Transporter	10	Transmembrane transporter activity; Long-chain fatty acid transport; Transmembrane transport; Long-chain fatty acid transporter activity; ATPase-coupled intramembrane lipid transporter activity; Transporter activity; ATPase-coupled transmembrane transporter activity; Amino acid/polyamine transporter I; Cationic amino acid transporter, C-terminal; Membrane transport proteins	***ABCD1, AQP3, ATP8B1, ATP8B2, ATP10D, ATP13A3, FABP5, PLIN2, SLC6A8, SLC7A4, SLC7A7, SLC13A5, SLC16A9, SLC16A10, SLC16A11, SLC22A3, SLC22A17, SLC27A1, SLC43A3, SLCO5A1, SPNS2***
Total	30	–	–

^*^Representative genes: Bold font = *P* < 0.05, Not bold = 0.05 ≤ *P* ≤ 0.1

^1^Keywords used to probe the list of pathways obtained from the lists generated with genes whose expression decreased between D10 and D14

^2^The number of pathways containing at least one of these keywords

^3^Name of all obtained pathways for each selected keyword

^4^Relative genes from obtained pathways containing at least one of these keywords

### Targeted analysis of select genes

We generated a list of 186 genes potentially involved in transport, catabolism, and synthesis of amino acids (see Additional file 1; Table S19) and a list of 133 genes was generated for lipids (see Additional file 1; Table S20). For amino acid-related genes, between D7 and D10, the expression of 56 genes increased and that of 80 genes decreased, while the expression of 50 genes remained constant. During this period, the expression of major amino-acid-catabolic enzymes (*ALDH3A2, CAD, DLD, ENO2/3, MTHFD1, GLUD1, ACMSD, PRODH, GCDH, FAH*) was decreased, suggesting attenuated intracellular degradation. Expression of genes coding for biosynthetic and inter-conversion enzymes (*ASL, PHGDH, PSAT1, ODC1, SMS*) was simultaneously suppressed, indicating reduced consumption of amino acids for new synthesis. In parallel, the expression of luminal secretory transporters (*SLC6A14, SLC36A2, SLC25A12, SLC25A15, SLC15A1, SLC46A1*) increased, potentially driving amino-acid efflux into the uterine lumen. Altogether, these changes are consistent with the accumulation of free amino acids in uterine fluid during this period.

Between D10 and D14, the expression of 48 genes increased and that of 31 genes decreased, while the expression of 107 genes remained constant. During this time, the expression of amino-acid catabolism-related genes was activated. For example, the expression of *BCKDHA, ACADS, ACAD8, DAO, GOT2, GPT2, ALDH2, MTHFR*, and *MCCC2* were increased, indicating intracellular degradation for energy and metabolite production. Concurrently, genes participating in amino acid-consuming pathways were induced (*ASL, ASS1, ARG1, ATIC, AGPAT5, PLOD2*), indicating the channeling of substrates into arginine/urea cycle intermediates and collagen synthesis. The expression of previously upregulated genes such as *HPD, PRODH, PHGDH*, and *ALDH18A1* was now decreased, apparently redefining the metabolic focus. Collectively, these changes are associated with intracellular consumption and reduced luminal efflux, resulting in decreased free amino acid concentrations in uterine fluid during this period (Table 6).

**Table 6:** Amino acid-related transcripts grouped according to the direction of change in expression and processes of synthesis, catabolism or transport

Process^1^	Direction of change in expression (D7 to D10)		Direction of change in expression (D10 to D14)	
	**Increased**^**2**^	**Decreased**^**3**^	**Increased**^**4**^	**Decreased**^**5**^
Synthesis	*AACS, ALDH2, ALDH4A1, ALDH5A1, ALDOB, GOT1, IDH1, NT5C3A, GATM, TAT, TKT, PSEN1, ALG14, ASS1, GAD1, GLS, GSS, GLUL, PSPH, PYCR1, ATP2B4, CKMT1A, AMD1, GLYCTK, LOC508879, RIMKLA, SDS*	*ALDH3A2, CAD, DLD, ENO2, ENO3, ENO4, FAHD1, FPGS, IDH2, LDHA, MTHFD1, NT5M, OGDH, PFKFB2, PFKM, ATP5MC1, GLUD1, PCCA, PPAT, MIF, NSD2, PRDM16, ALG8, CFAP20, TKTL1, CLN3, GCSH, GLYATL3, ALDH18A1, ASL, ODC1, PHGDH, PSAT1, SMS, CARNS1, CKB, GCLC, SAT1, MAT1A, MAT2A, ADSS2, MAT2B*	*ACO1, ACO2, ADPGK, ALDH2, ENO1, ALG3, ATP5F1B, ATP5ME, CTPS1, MDH2, MTHFR, NT5C, P4HA2, PGK1, PFKFB4, ASL, ASS1, ARG1, ATIC*	*PRDM6, ALDH18A1, PHGDH, CARNS1, SAT1, SAT2,*
Catabolism	*DAO, HPD, ADH6, DPYD, HADHA, HADHB, HMGCS1, ADH6, ENOPH1, MRI1, AASS, AGPAT2, BLMH, MTR, NOS3, TAT, EZH2, ATF4*	*ACMSD, CNDP1, CNDP2, SMOX, UROC1, ADHFE1, BDH1, FAH, GCDH, GGT1, HSD17B10, MMUT, OXCT1, PRODH, ADHFE1, ADO, GAT, GGT1, LAP3, MCCC2, UCP2, P4HA1, A0A3Q1NNL4, AFMID, ASRGL1, DBT, KYAT1, AHCYL1, AHCYL2, EZH1, EHMT2, KMT2A, LOC112441481, AASDH, HACD2, DNMT3B*	*BCKDHA, CNDP1, DAO, ACAD8, ACADS, AOC2, CHDH, HADHA, MCCC2, P4HA2, PLOD2, AGPAT5, ASRGL1, BCAT1, GOT2, GPT2, PAPSS1, EZH2, IDH3A*	*HPD, PRODH, AFMID, KYAT1, TPH1, MECOM, MGC138914*
Transport	*SLC6A14, SLC36A2, SLC25A12, SLC25A15, SLC11A1, SLC46A1, SLC39A2, SLC15A1*	*SLC3A2, SLC6A20, SLC6A9, SLC6A11, SLC7A4, SLC25A11, SLC25A13, SLC25A29, SLC39A8, SLC39A13, SLC25A22, SLC44A2*	*SLC7A8, SLC15A4, SLC36A2, SLC39A2, SLC43A2, SLC25A22, SLC44A2*	*SLC7A4, SLC7A7, SLC16A10, MFSD12*

^1^Processes regulating amino acid concentrations in the uterine lumen

^2^Genes whose expression increased from D7 to D10

^3^Genes whose expression decreased from D7 to D10

^4^Genes whose expression increased from D10 to D14

^5^Genes whose expression decreased from D10 to D14

For lipid-related genes, between D7 and D10, the expression of 58 genes increased and 48 genes decreased, while the expression of 27 genes remained constant. During this period, the expression of major lipid-catabolic enzymes (*LIPG, LPL, PLA2G15, PLA2G3, SMPD3, ABHD2, ACSL3/4, ELOVL7, DEGS2, SPHK1, SGMS2*) decreased while the expression of genes coding for biosynthesis (*ACACA, ACSL5, ACSL6, AGPAT2, PCYT1B, PTDSS1, SPTLC2, ELOVL2, UGCG)* increased. The expression of genes encoding for secretory phospholipases and lipid-transfer proteins (*GPLD1, PLA2G4A, PLA2G4F, GPCPD1, SLC27A1, SLC27A2, SLC27A5, NPC1L1, NPC2*) increased, while the expression of genes coding for re-uptake transporters (*SLC27A3, SLC27A4, SLC27A6, LRP1, APOE*) decreased. These changes in gene expression are consistent with elevated diglyceride and lipid concentrations in uterine fluid during this period.

Between D10 and D14, the expression of 32 genes increased and that of 21 genes decreased, while the expression of 80 genes remained constant. During this period, the expression of several lipid-catabolic genes remained decreased (*SMPD3, PLA2G2F, CYP27A1, SGPP1, LRAT, MBOAT2*). In parallel, expression of a different set of genes encoding secretory phospholipases and phosphatases (*GPLD1, PLA2G4F, DGKQ, GDPD3, PLD1, PLPP1, PLPP3*) was increased. Expression of genes involved in lipid synthesis (*FASN, ELOVL2, ELOVL4, ELOVL6, AGPAT5, DGAT1, CERS4, PCYT2, PTDSS2*) was also increased. The expression of genes coding for fatty-acid transporters shifted, with increased expression of *SLC27A2, SLC27A5, SLC27A6, ANGPTL8, LRP2* and *SLCO2A1*. These changes in gene expression are associated with the continuous increase in uterine luminal lipid concentrations during this period (Table 7).

**Table 7:** Lipid-related transcripts grouped according to the direction of change in expression and processes of synthesis, catabolism or transport

Process^1^	Direction of change in expression (D7 to D10)		Direction of change in expression (D10 to D14)	
	**Increased**^**2**^	**Decreased**^**3**^	**Increased**^**4**^	**Increased**^**5**^
Synthesis	*GPLD1, PLA2G4F, ACER2, AGK, DGKE, DGKK, GPCPD1, INPP1, IP6K2, PIK3R3, PLCD1, PLCE1, PLCH1, PLCH2, SIRT6, SYNJ1, SPTLC2, ELOVL2, DEGS1, ACACA, ACSL5, ACSL6, AGPAT2, PCYT1B, PTDSS1, SQLE, UGCG*	*NSDHL, DGKQ, DGKI, ITPKA, PIP4K2A, CHKA, DGKA, ITPR1, ITPR3, PI4KA, PIK3CB, PLCG1, PRDM16, PTEN, SIRT1, SIRT3, SIRT7, PCYT2, LRAT, MBOAT2, ACSL3, ACSL4, DEGS2, ELOVL7, HACD2, PANK1, SGMS2, SMS, SPHK1*	*GPLD1, PLA2G4F, DGKQ, GDPD3, PLD1, PLPP1, PLPP3, SPTLC2, ELOVL2, DEGS1, PCYT2, AGPAT5, CERS4, DGAT1, ELOVL4, FASN, PTDSS2*	*ACER2, G6PD, GPD1L, PLCB1, DGKI, ITPKA, PIP4K2A, NSDHL, CERS6, ETNK2, PANK3, LRAT, MBOAT2*
Catabolism	*HADHA, HADHB, LIPA, ACAT1, ACAT2, ACSF3, LPAR6, CYP24A1, ALOX12, ALOX12B, ALOX15B, BCO1, CYP11A1, CYP51A1, LCAT, LPIN2, PLA2G4A, PTGS2, SC5D, SCD5, SGPL1, SOAT1, ZDHHC9*	*SMPD3, ABHD2, LIPG, LPL, PLA2G15, PLA2G3, PTGIS, TECR, TM7SF2*	*HADHA, ACOT8, PAFAH2, LPAR6, CYP24A1, ACER1, PTGES2, PTGS1*	*SMPD3, PLA2G2F, CYP27A1, SGPP1, PTGIS*
Transport	*SLC27A2, SLC27A5, LPAR1, LRPAP1, SLC27A1, NPC1L1, NPC2, SLCO3A1*	*ANGPTL8, LRP1, APOE, LDLRAP1, SLC27A3, SLC27A4, TSPO, SLC4A4, SLC27A6*	*SLC27A2, SLC27A5, SLC27A6, ANGPTL8, LRP2, SLC4A4, SLCO2A1*	*SLC27A1, LRP1*

^1^Processes regulating lipid concentrations in the uterine lumen

^2^Genes whose expression increased from D7 to D10

^3^Genes whose expression decreased from D7 to D10

^4^Genes whose expression increased from D10 to D14

^5^Genes whose expression decreased from D10 to D14

## Discussion

The uterine metabolome is composed of molecules from several biochemical classes, including amino acids and lipids. For example, amino acids may serve as energy substrates, osmolytes, and precursors for protein synthesis, whereas lipids are involved in membrane biogenesis, participate in paracrine signaling, and are involved in the remodeling of the endometrium during the estrous cycle. Here, we applied targeted metabolomics and transcriptomics to test the hypothesis that the concentrations of amino acids and lipids in the uterine luminal fluid, and the gene expression in luminal epithelial cells, change across the second week of the estrous cycle. Altogether, we determined that while concentrations of amino acids and lipids increased from D7 to D10, amino acid concentrations decreased, while that of lipids continued to increase from D10 to D14 (Table 1 and Additional file 1; Table S1). This suggested to us that there are specific regulatory mechanisms in the luminal epithelium that selectively control luminal abundance of metabolites, according to the biochemical class. Indeed, luminal epithelial cell transcriptome and functional enrichment analysis indicated genes and processes that changed temporally according to changes in the metabolome. Target genes whose expression changed temporally have documented roles in transport, catabolism, and synthesis of amino acids and lipids. Other authors have documented metabolite and transcript changes in the uterus, but they have focused on different time windows, used whole endometrium for gene expression and whole uterine lumen washes post-mortem and have collected a single sample per animal [17–20, 35, 40, 41]. In the current report, for the first time, we collected luminal epithelial cells and uterine fluid and conducted analysis on the same samples from the same cows during this time window. Systemic regulation, such as that exerted by progesterone, likely controls the luminal metabolome during the second week post-estrus. Indeed, previous studies have shown that concentrations of amino acids, carbohydrates and lipids in the uterine luminal fluid change over time and are modulated by circulating serum progesterone levels [17, 20].

Multivariate metabolomic analyses revealed dynamic regulation of metabolites between D7, D10, and D14 of the estrous cycle, driven mainly by shifts in amino acids and lipids. Supervised multivariate approaches (PLS-DA and OPLS-DA) clearly separated the sampling days, indicating biologically meaningful temporal differences. Variable importance in projection (VIP) analysis identified phospholipids, amino acids (glutamine, arginine, ornithine), polyamines (putrescine), sphingolipids, and cholesterol esters as the primary drivers of the temporal separation. Metabolite set enrichment and pathway impact analyses highlighted amino acid metabolism (methionine, glycine–serine, glutathione, beta-alanine, glutamate, aspartate, histidine, alanine, arginine–proline) and lipid biosynthetic pathways (phosphatidylcholine, phosphatidylethanolamine, and sphingolipid metabolism) as over-represented functional categories (Figs. 2, 3 and 4). Similar results were reported by Silva et al. [35]. They documented a greater concentration of molecules in the uterine lumen and epithelial cell expression of genes involved in the sphingolipid and choline metabolism pathways at D14 of the estrous cycle. The above-mentioned pathways provide precursors for glandular growth, extracellular matrix remodeling, polyamine synthesis, and membrane production. The untargeted approach for transcriptomic analysis also revealed distinct temporal expression patterns. Between D7 and D10, gene expression changes were related to increased secretion, energy production, proliferation, and signaling pathways. Concurrently, reduced uptake and catabolism of amino acids and lipids promoted the accumulation of metabolites within the uterine lumen. Between D10 and D14, gene expression changes indicated increased lipid synthesis, amino acid catabolism, and membrane transport while reducing protein degradation, insulin signaling, and hormonal integration (see Additional file 1; Tables S15–S18). This suggests that histotroph secretions are continuously increased along with the increase in lipid and protein content in the uterine lumen during the luteal phase with enhanced nutrient signaling.

Eleven amino acids, including both essential (arginine, glutamine, histidine, tryptophan and valine) and non-essential (alanine, asparagine, aspartic acid, glutamic acid, glycine and serine), showed temporal shifts from D7 to D14 (Fig. 5A). Amino acids serve multiple critical roles such as providing mitogenic signals, directing protein synthesis, acting as energy substrates, controlling the uterine oxidative status, regulating gene expression and modulating the immune system [31]. The calculated metabolite variables highlighted coordinated regulation of the amino acid pool across the estrous cycle. The increased concentration of essential amino acids between D7 and D10, and the decrease in the ratio of non-essential to essential amino acids from D7 to D14 reflect the disproportionate consumption or reabsorption of specific essential amino acids (Fig. 5B). Moreover, keyword-based analysis of transcriptomics data for enriched pathways confirmed that amino acid-related pathways (i.e., arginine/proline metabolism, glycine/serine/threonine metabolism) were extensively represented during the time window in this study (Tables 2, 3, 4 and 5). This suggests that luminal amino acid changes are likely driven by gene regulation in the endometrial epithelial cells. Additionally, transcript-level analysis revealed dynamic regulation of synthesis, catabolism, and transport genes for amino acids. Between D7 and D10, gene expression of amino acid catabolic enzymes (*ALDH3A2, CAD, DLD, MTHFD1, GLUD1, ACMSD, PRODH, GCDH, FAH*) and biosynthetic enzymes (*ASL, PHGDH, PSAT1, ODC1, SMS*) decreased while transcription of secretory transporters (*SLC6A14, SLC36A2, SLC25A12, SLC25A15, SLC15A1, SLC46A1*) increased, driving luminal accumulation. From D10 to D14, expression of catabolic enzymes (*BCKDHA, ACADS, ACAD8, DAO, GOT2, GPT2, ALDH2, MTHFR*) and uptake transporters (*SLC7A8, SLC43A2, SLC36A2, SLC15A4, SLC25A22, SLC44A2*) was induced (Table 6 and Additional file 1: Table S19). We speculate that these changes could lead to intracellular utilization of amino acids for protein synthesis, thereby lowering luminal amino acid concentrations. Previous research also documented a strong correlation between mRNA expression of amino acid transporters in endometrial cells and their respective concentrations in luminal secretions [19, 27]. This time-dependent regulation of amino acids in the uterine luminal fluid and transcripts in the epithelial cells seem to be a relevant feature of uterine function of the estrous cycle. It is tempting to speculate that they play a role to support pregnancy.

Multiple lipid classes, including cholesterol esters, glycosylceramides, sphingolipids, glycerophospholipids, and triacylglycerols, displayed concentration changes across time (Figs. 6A, C, 7A, C, and 8A). The continuous lipid enrichment from D7 to D14 likely supports membrane biogenesis, provides precursors for lipid-mediated signaling (ceramides, sphingosine-1-phosphate), establishes an energy-dense reservoir for the rapidly elongating conceptus, and contributes to the proliferation and reorganization of trophectoderm cells [29]. These results were similar as reported by Silva et al. [35] where they documented that the concentrations of sphingolipids, glucosylceramides, glycerophospholipids, and ceramides were greater in the uterine luminal metabolome at D14 compared to D4 and D7, and by King et al. [42] where they reported that the concentration of long-chain fatty acids and oxylipins were greater at D15 compared to D5 and D10. For the calculated metabolite variables of lipids, sums of glycerophospholipids, sphingomyelins, and triacylglycerols increased steadily, whereas the diacyl-phosphatidylcholine:choline and phosphatidylcholine:choline ratios decreased from D7 to D10 before recovering by D14, suggesting transient activation of phospholipase activity or altered choline recycling. Elevated sums of long-chain and polyunsaturated fatty acid-containing lipids, together with specific ceramide and hexosylceramide species, are recognized modulators of cellular proliferation, apoptosis, and embryo-endometrial crosstalk (Figs. 6B, D, 7B, D, 8B). However, specific mechanisms are unknown. Keyword-based analysis of transcriptomics data for enriched pathways confirmed that lipid-related pathways (i.e., sphingolipid metabolism) were represented during the study, which suggests that luminal lipid variations are likely driven by gene regulation in the endometrial epithelial cells (Tables 2, 3, 4 and 5). Moreover, transcript-level analysis revealed dynamic regulation of synthesis, catabolic, and transport genes for lipids. Between D7 and D10, lipid catabolic enzymes (*LIPG, LPL, PLA2G15, PLA2G3, SMPD3, ABHD2, ACSL3, ACSL4*) decreased while biosynthetic enzymes (*ACACA, ACSL5, ACSL6, AGPAT2, PCYT1B, PTDSS1, SPTLC2*) and secretory transporters (*GPLD1, PLA2G4A, PLA2G4F, GPCPD1, SLC27A1, SLC27A2, SLC27A5*) increased and re-uptake of transporters (*SLC27A3, SLC27A4, SLC27A6, LRP1, APOE*) decreased, potentially driving luminal lipid accumulation. From D10 to D14, catabolism remained suppressed, a new set of biosynthetic enzymes (*FASN, ELOVL2, ELOVL4, ELOVL6, AGPAT5, DGAT1*) and secretory enzymes (*GPLD1, PLA2G4F, DGKQ, GDPD3, PLD1, PLPP1, PLPP3*) were induced, and transporter expression shifted to support continued lipid enrichment in the uterine lumen (Table 7 and Additional file 1; Table S20). Previous research reported an increased concentration of metabolites of the sphingolipid metabolism pathway in addition to increased expression of enzymes responsible for their synthesis [35]. The temporal reprogramming in the bovine uterine secretory cycle links transcriptomic shifts in secretory gene expression to metabolomic profiles in luminal fluid.

## Conclusion

**Fig. 10:**
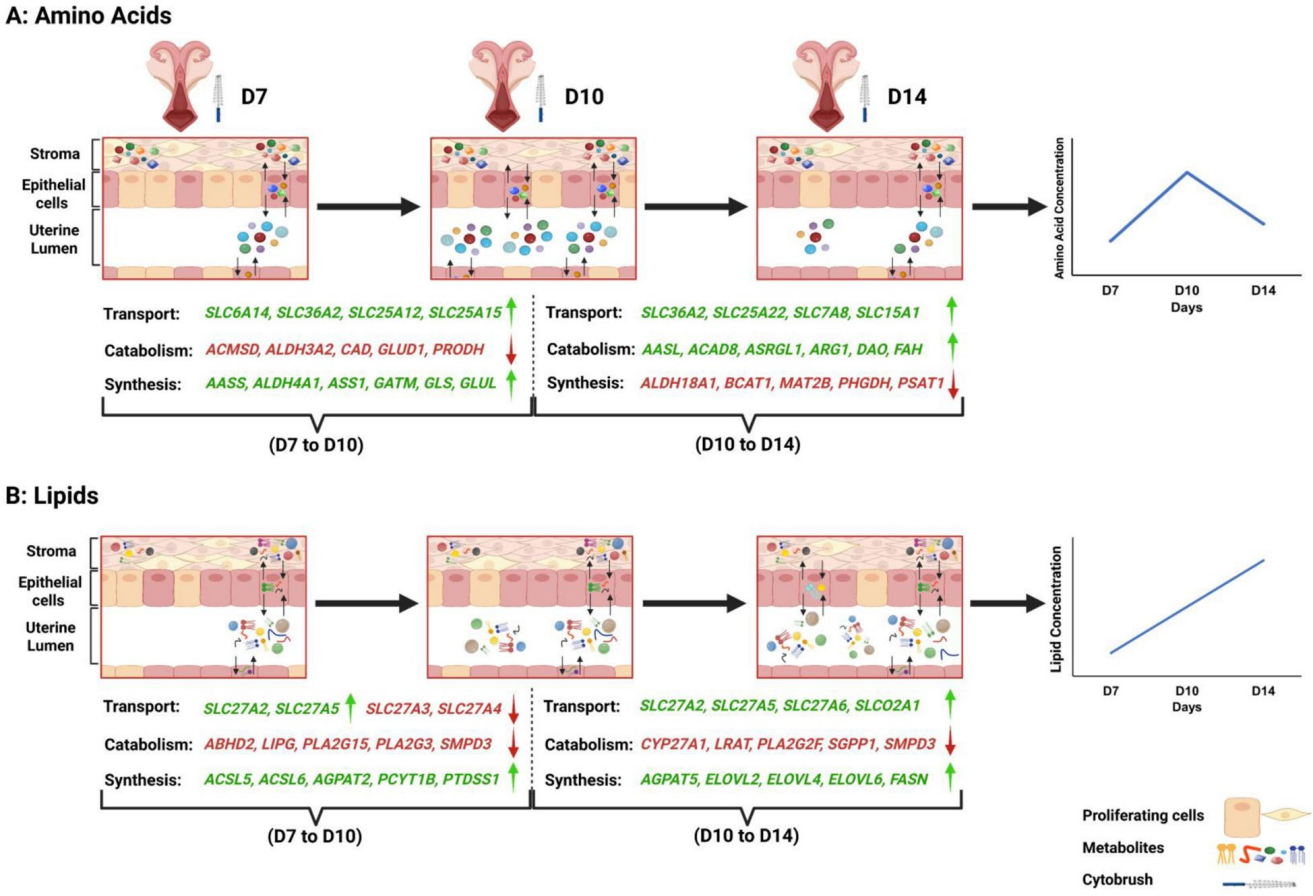
Hypothetical model of the molecular control of temporal changes in amino acid (**A**) and lipid (**B**) concentrations in the uterine lumen 7, 10, and 14 d after estrus. **A** From D7 to D10, increased expression of amino acid transporters and biosynthetic genes and reduced expression of amino acid catabolism genes promote accumulation of free amino acids in the uterine lumen. From D10 to D14, enhanced expression of amino acid transport and catabolic genes and reduced amino acid biosynthetic capacity coincide with decreased luminal amino acid concentrations. **B** Between D7 and D10, elevated expression of lipid transport and synthesis genes, coupled with reduced lipid catabolism and reduced re-uptake transport, supports rising luminal lipid content. From D10 to D14, sustained upregulation of lipid transporters and biosynthetic enzymes, along with continued suppressed expression of lipid catabolic genes, drives further lipid accumulation in the uterine lumen. Line plots depict overall temporal trends in luminal amino acid and lipid concentrations. Green arrows indicate increased gene expression, red arrows indicate decreased gene expression. Arrows within cells denote metabolite transport

We reported the dynamic shifts in amino acid and lipid metabolites in the uterine luminal fluid, supported by transcriptomic changes from epithelial cells. The changes in the luminal concentration of metabolites could be due to increased synthesis, degradation and bi-directional transport of nutrients between lumen and epithelium (Fig. 10). Nevertheless, these hypothetical regulatory mechanisms require further investigation for functional validation of key metabolites/genes and the understanding of the implications for fertility management and embryo survival.

## Supplementary Information

Additional file 1: Table S1. Changes across time points in the concentrationof all metabolites in uterine luminal fluid. Table S2. Changes across time points in significant calculated variables. Table S3. Detail of all genes in pattern A. Table S4. Detail of all genes in pattern B. Table S5. Detail of all genes in pattern C. Table S6. Detail of all genes in pattern D. Table S7. Detail of all genes in pattern E. Table S8. Detail of all genes in pattern F. Table S9. Detail of all genes in pattern G. Table S10. Detail of all genes in pattern H. Table S11. Detail of all genes in pattern I, encompassing patterns A, B, and D. Table S12. Detail of all genes in pattern J, encompassing patterns D, E, and G. Table S13. Detail of all genes in pattern K, encompassing patterns A, C, and H. Table S14. Detail of all genes in pattern L, encompassing patterns E, F, and H. Table S15. Top 50 enriched terms for pattern Iby significant FDR ≤ 0.1 to evaluate the transcriptomic time trends. Table S16. Top 50 enriched terms for pattern Jby significant FDR ≤ 0.1 to evaluate the transcriptomic time trends. Table S17. Top 50 enriched terms for pattern Kby significant FDR ≤ 0.1 to evaluate the transcriptomic time trends. Table S18. Top 50 enriched terms for pattern Lby significant FDR ≤ 0.1 to evaluate the transcriptomic time trends. Table S19. Classification of amino acid-related genes according to their main function and direction of change over time. Table S20. Classification of lipid-related genes according to their main function and direction of change over time

Additional file 2: Table S21. RNA samples quality control results

Additional file 3: Fig. S1. Summary of the steps taken on the transcriptomic untargeted analysis, for the generation of data lists and tables. Fig. S2. Summary of the steps taken on the transcriptomic targeted analysis, for the generation of data lists and tables. Fig. S3. Temporal patternsof gene expression from D7 to D14. *n* = Number of genes in each pattern that were significantly affected by timeor showed a tendency for time effect.

## Data Availability

All data generated or analyzed during this study are included in this published article and its supplementary information files. Further datasets used and/or analysed during the current study are available from the corresponding author on reasonable request.

## Abbreviations

ABC: ATP-binding cassette transporter
ESR: Estrogen receptor
GPR40: G-protein coupled receptor 40
IFNT: Interferon-tau
ISG: Interferon-stimulated gene
LC-MS: Liquid Chromatography-Mass Spectrometry
LE: Luminal epithelial cells
OXTR: Oxytocin receptor
PCA: Principal component analysis
PGR: Progesterone receptor
PGF_2_α: Prostaglandin F_2_α
PI3K/AKT: Phosphatidylinositol 3-kinase (PI3K)-Akt signaling pathway
PLS-DA: Partial least squares discriminant analysis
PVDF: Polyvinylidene fluoride
SLC: Solute carrier transporter
ULF: Uterine luminal fluid
VIP: Variables important for projection
