# Supplementary material for: Metabolomic and transcriptomic landscape of the bovine uterine lumen during the second week of the estrous cycle
Source: J Anim Sci Biotechnol. 2026 Jul 9;17:143. doi: 10.1186/s40104-026-01466-z (PMC13348268; doi:10.1186/s40104-026-01466-z)
Supplement: Supplementary file 3 — Additional file 3: Fig. S1. Summary of the steps taken on the transcriptomic untargeted analysis, for the generation of data lists and tables. Fig. S2. Summary of the steps taken on the transcriptomic targeted analysis, for the generation of data lists and tables. Fig. S3. Temporal patternsof gene expression from D7 to D14. n = Number of genes in each pattern that were significantly affected by timeor showed a tendency for time effect. [file 40104_2026_1466_MOESM3_ESM.pdf]

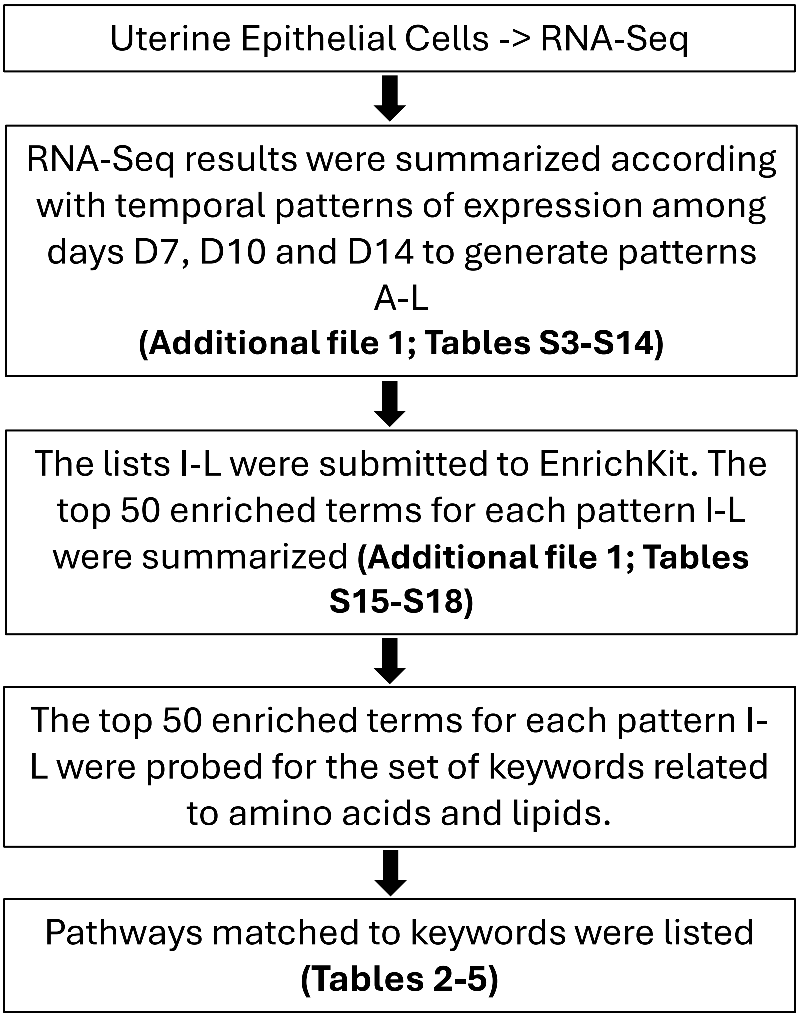
Uterine Epithelial Cells -> RNA-Seq
RNA-Seq results were summarized according with temporal patterns of expression among days D7, D10 and D14 to generate patterns A-L
(Additional file 1; Tables S3-S14)
The lists I-L were submitted to EnrichKit. The top 50 enriched terms for each pattern I-L were summarized (Additional file 1; Tables S15-S18)
The top 50 enriched terms for each pattern I-L were probed for the set of keywords related to amino acids and lipids.
Pathways matched to keywords were listed
(Tables 2-5)

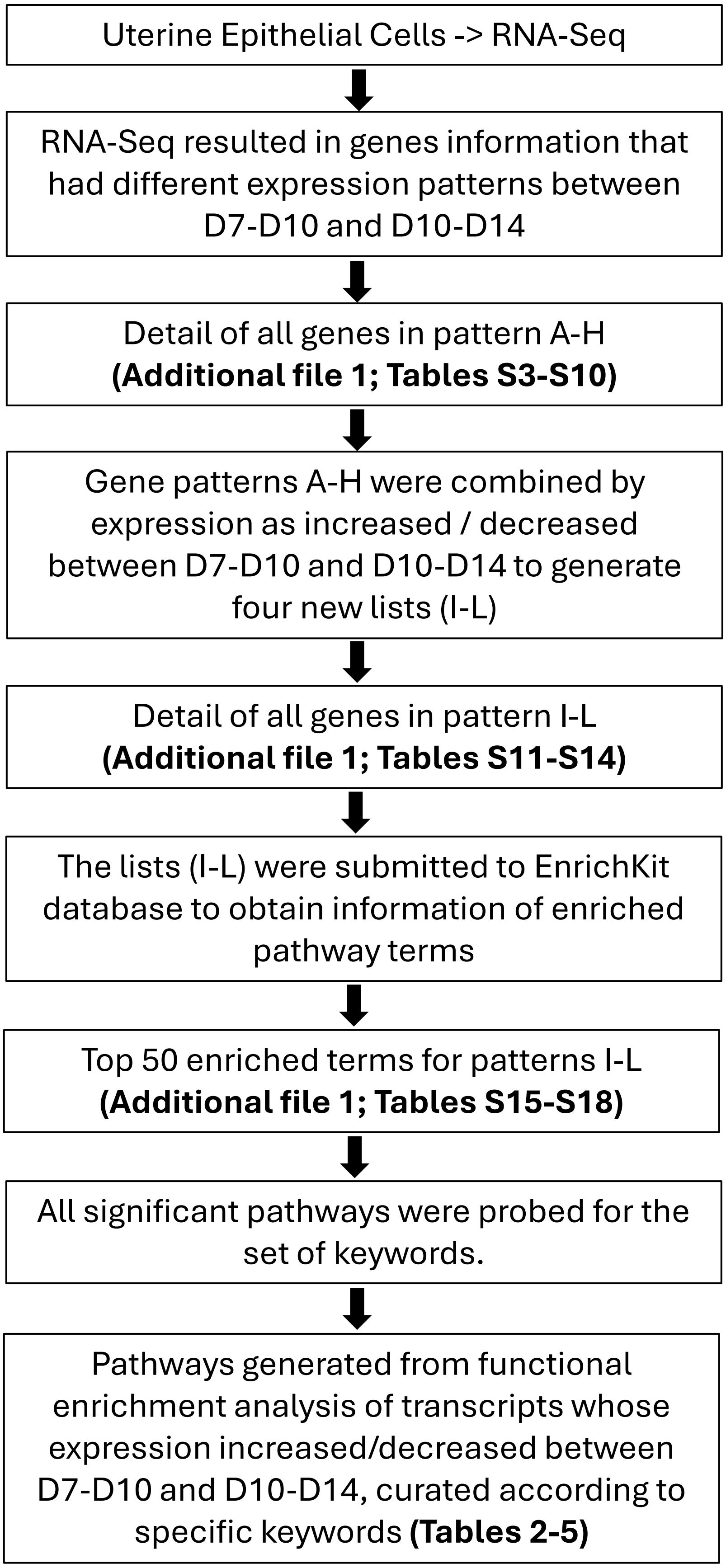
Uterine Epithelial Cells -> RNA-Seq
RNA-Seq resulted in genes information that had different expression patterns between D7-D10 and D10-D14
Detail of all genes in pattern A-H
(Additional file 1; Tables S3-S10)
Gene patterns A-H were combined by expression as increased / decreased between D7-D10 and D10-D14 to generate four new lists (I-L)
Detail of all genes in pattern I-L
(Additional file 1; Tables S11-S14)
The lists (I-L) were submitted to EnrichKit database to obtain information of enriched pathway terms
Top 50 enriched terms for patterns I-L
(Additional file 1; Tables S15-S18)
All significant pathways were probed for the set of keywords.
Pathways generated from functional enrichment analysis of transcripts whose expression increased/decreased between D7-D10 and D10-D14, curated according to specific keywords **(Tables 2-5)**

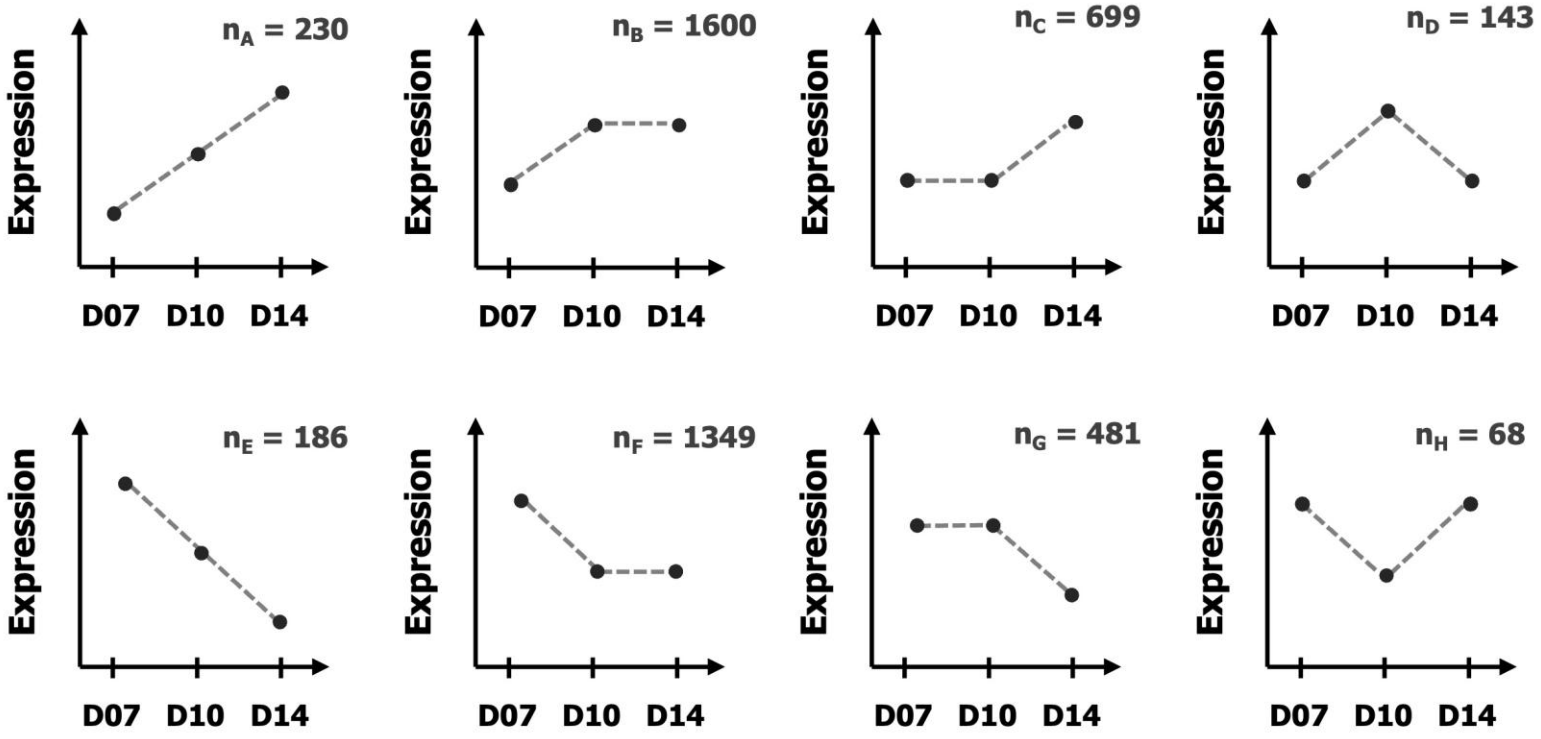

$n_A = 230$
$n_B = 1600$
$n_C = 699$
$n_D = 143$
$n_E = 186$
$n_F = 1349$
$n_G = 481$
$n_H = 68$
Expression
D07
D10
D14
